# Network enrichment significance testing in brain–phenotype association studies

**DOI:** 10.1002/hbm.26714

**Published:** 2024-06-15

**Authors:** Sarah M. Weinstein, Simon N. Vandekar, Bin Li, Aaron F. Alexander‐Bloch, Armin Raznahan, Mingyao Li, Raquel E. Gur, Ruben C. Gur, David R. Roalf, Min Tae M. Park, Mallar Chakravarty, Erica B. Baller, Kristin A. Linn, Theodore D. Satterthwaite, Russell T. Shinohara

**Affiliations:** ^1^ Department of Epidemiology and Biostatistics Temple University College of Public Health Philadelphia Pennsylvania USA; ^2^ Department of Biostatistics Vanderbilt University Medical Center Nashville Tennessee USA; ^3^ Department of Computer and Information Sciences Temple University College of Science and Technology Philadelphia Pennsylvania USA; ^4^ Department of Psychiatry University of Pennsylvania, Perelman School of Medicine Philadelphia Pennsylvania USA; ^5^ Department of Child and Adolescent Psychiatry and Behavioral Science Children's Hospital of Philadelphia Philadelphia Pennsylvania USA; ^6^ Section on Developmental Neurogenomics National Institute of Mental Health Intramural Research Program Bethesda Maryland USA; ^7^ Department of Biostatistics, Epidemiology, and Informatics University of Pennsylvania, Perelman School of Medicine Philadelphia Pennsylvania USA; ^8^ Department of Psychiatry, Temerty Faculty of Medicine University of Toronto Toronto Ontario Canada; ^9^ Integrated Program in Neuroscience McGill University QC Canada; ^10^ Department of Psychiatry McGill University QC Canada; ^11^ Cerebral Imaging Centre, Douglas Research Centre, McGill University QC Canada

**Keywords:** brain networks, brain‐phenotype associations, enrichment, hypothesis testing

## Abstract

Functional networks often guide our interpretation of spatial maps of brain–phenotype associations. However, methods for assessing enrichment of associations within networks of interest have varied in terms of both scientific rigor and underlying assumptions. While some approaches have relied on subjective interpretations, others have made unrealistic assumptions about spatial properties of imaging data, leading to inflated false positive rates. We seek to address this gap in existing methodology by borrowing insight from a method widely used in genetics research for testing enrichment of associations between a set of genes and a phenotype of interest. We propose network enrichment significance testing (NEST), a flexible framework for testing the specificity of brain–phenotype associations to functional networks or other sub‐regions of the brain. We apply NEST to study enrichment of associations with structural and functional brain imaging data from a large‐scale neurodevelopmental cohort study.

## Graphical abstract

Functional networks often inform how we interpret spatial maps of brain–phenotype associations, yet existing methods rely on strong assumptions and may not be replicable. To address this gap in existing methodology, we propose a new method, called NEST.

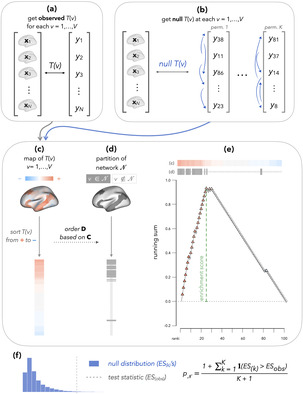

## 1 INTRODUCTION

Quantifying and spatially mapping brain–phenotype associations is a central component of many neuroimaging studies. There is often particular interest in interpreting these maps through the lens of canonical functional networks, which delineate areas of the brain known to participate in different behaviors and cognitive functions (Schaefer et al., 2018; Yeo et al., 2011). Evaluating whether brain–phenotype associations are especially strong—or *enriched*—within a network of interest can add to our understanding of the neural mechanisms underlying transdiagnostic psychopathology (Segal et al., 2023).

Recent methods in network neuroscience have made it possible to test different properties of brain networks (Váša & Mišić, 2022). Still, methods specifically for evaluating enrichment of brain–phenotype associations within networks have varied. For example, some researchers have used descriptive statistics or parametric tests to assess differences in mean brain–phenotype correlations found inside versus outside a network, which do not account for complex distributional features of brain imaging data. Subjective interpretations—such as visualizing a map of brain–phenotype associations beside a network partition map and noting any networks with which strong associations appear to coincide—are also quite common. While such claims are often compelling, the reliance on descriptive or subjective methods, rather than statistical methods with well‐defined null hypotheses, may compromise generalizability.

Some researchers have indeed shifted toward more methodical assessments of brain–phenotype association maps in relation to functional networks by using the *spin test*. Building on earlier work by Vandekar et al. (2015) and Gordon et al. (2016), the spin test (Alexander‐Bloch et al., 2018) evaluates spatial alignment between two maps through a spatial null modeling procedure that compares an observed measure of spatial correspondence between two maps to a null distribution of spatial correspondence, obtained by projecting cortical surface map onto a sphere and randomly rotating (i.e., “spinning”) it. Several adaptations and extensions of the method have also been described (Cornblath et al., 2020; Váša et al., 2018; Vázquez‐Rodríguez et al., 2019), and this framework has been employed in numerous settings to test claims about network alignment with maps of brain–phenotype associations (Baller et al., 2022; Baum et al., 2019; Hu et al., 2022; Mandal et al., 2020; Markello et al., 2022; Nadig et al., 2021; Petersen et al., 2022).

However, the spin test has also been subjected to a fair amount of criticism. One concern is that the method's underlying null hypothesis may not be an appropriate translation of the scientific hypotheses it is often used to test (Pang et al., 2023). The spin test evaluates spatial alignment between two maps, and it is plausible that it might yield a statistically significant result if a map of brain–phenotype associations contained greater spatial smoothness—but not necessarily stronger associations—within versus between networks. Several other limitations of the spin test have been noted, including that it is applicable only to surface‐based (not volumetric) data and that it requires assuming covariance stationarity for statistical inference to be generalizable across datasets (Burt et al., 2020; Markello & Misic, 2021; Weinstein et al., 2021). Váša and Mišić (2022) also highlight that the spin test may be sensitive to the choice of algorithm used for rotations (e.g., random spherical rotations around axis in three‐dimensional space, as in Alexander‐Bloch et al., 2018, versus using uniformly distributed rotation matrices obtained via QR decomposition, as in Lefèvre et al., 2018).

Another approach was described by Park et al. (2018) in a study of enrichment of associations between brain structure and neuropsychiatric diagnoses. These authors borrowed insight from gene set enrichment analysis (GSEA) (Subramanian et al., 2005), which has been widely used in genetics research to test enrichment of sets of genes for particular phenotypes—for example, to examine the collective role of a group of genes (rather than individual genes) on disease risk. Park et al. (2018) specifically adopted a variation of GSEA, known as FastGSEA (Korotkevich et al., 2016). Both GSEA and FastGSEA involve computing an “enrichment score,” which quantifies the relative strength of gene–phenotype associations for genes found within versus outside a pre‐specified set of genes, using permutations to derive a null distribution. The main difference between these two approaches lies in the unit of permutation: while GSEA permutes samples before re‐computing the enrichment score to form a null distribution, FastGSEA permutes gene labels. The translation of FastGSEA to neuroimaging involves permuting network labels in a manner that destroys the underlying spatial structure of the data, violating the exchangeability assumption essential for type I error control in permutation tests.

In this article, we propose network enrichment significance testing (NEST), adapting GSEA by Subramanian et al., 2005. The proposed method operationalizes the notion of enrichment through a statistic which quantifies the degree to which associations found inside the network are more extreme than those found outside the network. As we will illustrate, NEST controls type I error rates by permuting participants in order to construct a suitable null distribution, instead of permuting spatial units (e.g., vertices), which would require making implausible assumptions about the spatial structure of the data.

The remainder of this article is organized as follows. In Section 2, we define the underlying null hypothesis and implementation of our proposed method. In Section 3, we apply and compare our method with previous approaches in studies of network enrichment of brain–phenotype associations using structural and functional MRI data from the Philadelphia Neurodevelopmental Cohort. Finally, in Section 4, we discuss implications, limitations, and future directions of our method.

## 2 METHODS

### 2.1 Null hypothesis

**FIGURE 1:**
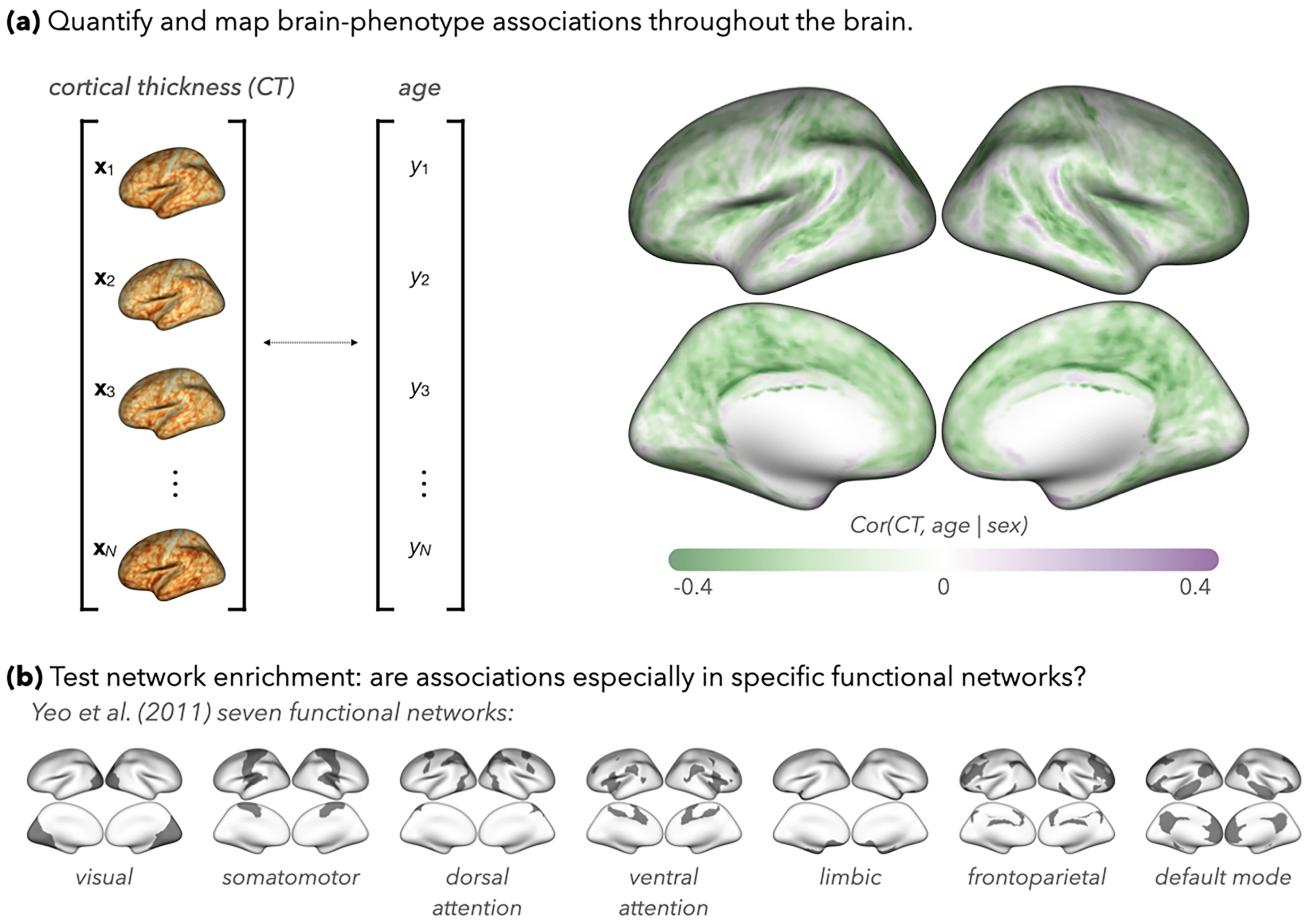
Example of how brain–phenotype associations are (a) quantified and spatially mapped and (b) compared to maps of functional networks (e.g., those delineated by Yeo et al., 2011). As we discuss in Section 1, existing methods for evaluating network specificity (or “enrichment”) of brain–phenotype associations have been subjective, which may preclude reproducibility, or relied on strong assumptions, which may result in type I error inflation. In Section 2, we propose a new approach, called NEST, to address these limitations.

Let $T\left(v\right)$ denote a statistic of brain–phenotype associations (e.g., correlation or a metric derived from a regression model), computed for a given image location v (v=1,…,V), using data across N participants (e.g., as in Figure 1a). In this article, we consider brain measurements and annotation maps on the cortical surface, with v denoting a vertex on the surface; however, the same framework can be generalized to volumetric data. Let $N\subseteq \left\{1,\ldots ,V\right\}$ denote a subset of all vertices indexing a network or other region of interest which has been prespecified by the researcher (e.g., from Figure 1b). Finally, let $T_{N}$ denote a random variable whose realizations, $T\left(v\right)$, are found at randomly selected locations inside the network ($T\left(v\right)$ for v∈N), and let $T_{N^{c}}$ denote a random variable whose realizations are found at locations outside the network ($T\left(v\right)$ for v∉N). Here, we define network enrichment to occur when realizations of $T_{N}$ tend to be more extreme (i.e., have higher magnitude) than realizations of $T_{N^{c}}$. In the current definition of network enrichment, we assume that enrichment is sign‐consistent; that is, within an enriched network, realizations of $T_{N}$ tend to be positively correlated. We also assume that the set of vertices in the network, N, has been pre‐specified (e.g., from previous literature such as Yeo et al., 2011).

We propose testing enrichment by comparing the stochastic ordering of these random variables. For two random variables Y and Z, if

(1) $$\mathrm{Pr}\left(Y>w\right)\geq \mathrm{Pr}\left(Z>w\right)\;\forall \;w\in \left(-\infty ,\infty \right)$$

then Y is stochastically greater than Z (or $Y{≽}_{\mathrm{st}}Z$) and “strictly” stochastically greater (or $Y{≻}_{\mathrm{st}}Z$) if there exists a w such that $\mathrm{Pr}\left(Y>w\right)>\mathrm{Pr}\left(Z>w\right)$ (Belzunce et al., 2015). We consider a variation of this concept for the current setting, where we are interested in characterizing stochastic ordering in terms of the “extremeness” of realizations of the $T_{N}$ and $T_{N^{c}}$. Here, we characterize the random variable Y to be “stochastically more extreme” than Z if either of the following (2 or 3) is true:

(2) $$\mathrm{Pr}\left(Y>w\right)\geq \mathrm{Pr}\left(Z>w\right)\;\forall \;w\in \left[0,\infty \right),$$

or

(3) $$\mathrm{Pr}\left(Y<w\right)\geq \mathrm{Pr}\left(Z<w\right)\;\forall \;w\in \left(-\infty ,0\right],$$

and, additionally, if either (2) or (3) holds strictly for some w. We denote this relationship with the following notation:

(4) $$Y{≻}_{\mathrm{ex}}Z.$$

Finally, our proposed null hypothesis for testing enrichment in a given network N is

(5) $$H_{0}:T_{N}{⊁}_{\mathrm{ex}}T_{N^{c}},$$

so that when $H_{0}$ is true, $T_{N}$ is “stochastically no more extreme” than $T_{N^{c}}$. Under the alternative hypothesis, $T_{N}{≻}_{\mathrm{ex}}T_{N^{c}}$.

As we will discuss in the next section, to test the proposed $H_{0}$, we use a test statistic called an enrichment score (ES). Briefly, the ES captures the distance between the distributions of $T_{N}$ and $T_{N^{c}}$, without making assumptions about distributional features of either random variable. Thus, the ES quantifies the degree to which realizations of $T_{N}$ are more extreme than realizations of $T_{N^{c}}$; therefore, under the alternative hypothesis, the ES would be larger, and under the null hypothesis, the ES would be smaller.

### 2.2 Network enrichment significance testing (NEST)

For a given brain–phenotype association (e.g., cortical thickness, age) and pre‐specified set of network indices $N\subseteq \left\{1,\ldots ,V\right\}$, we propose the following adaptation of Subramanian et al.'s (2005) GSEA for NEST.

**FIGURE 2:**
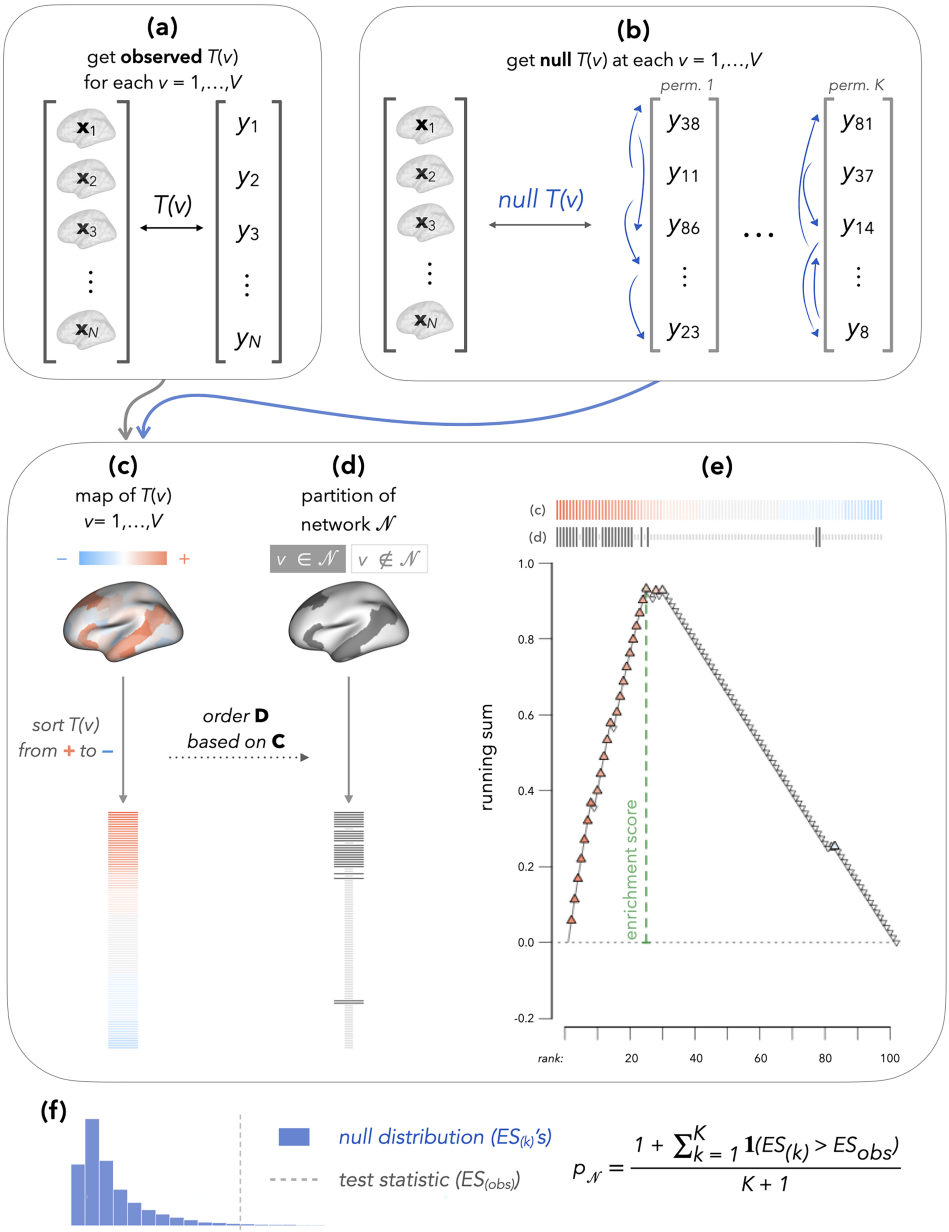
Illustration of NEST, which adapts Subramanian et al.'s (2005) GSEA to test network enrichment in brain–phenotype association studies. In (a), we illustrate how local brain–phenotype associations are quantified at each vertex using a statistic, $T\left(v\right)$, which we compute at every vertex (v=1,…,V). In (b), we show that by permuting participants, we obtain a null version of $T\left(v\right)$ (also computed for v=1,…,V). In (c), we sort the values of $T\left(v\right)$ from positive to negative and in (d), we match the order of the sorted list of $T\left(v\right)$ to the binary network annotation map, which partitions the brain into locations inside and outside N. In (e), we obtain our test statistic (enrichment score, ES), which quantifies the extent to which values of $T\left(v\right)$ with larger magnitude tend to appear within versus outside the network N. A running sum is initialized at 0 and increases by an increment proportional to $T\left(v\right)$ for v∈N and decreases by a uniform increment (proportional to the number of v∉N) otherwise. The ES (green dotted line) is defined as the largest magnitude attained by the running sum over the entire progression down the list. We calculate the ES based on both observed $T\left(v\right)$s (those obtained in a) and null $T\left(v\right)$s (those obtained in b), and in (f), we estimate a p‐value by comparing the observed and null enrichment scores.

1. Quantify brain–phenotype associations $T\left(v\right)$ at each location (v=1,…,V). The choice of metric is up to the researcher (e.g., partial correlation, as in Figure 1b, regression coefficient estimates, Wald statistic, or something else), but it must have a sign.
2. Sort the $T\left(v\right)$ in descending order, so that the v's with the strongest positive brain–phenotype associations appear at the top of the list, and vertices with strongest negative associations appear at the bottom of the list (Figure 2a). In the ranked list, we denote the jth entry as $T\left(v_{\left(j\right)}\right)=$ (equal to the jth largest $T\left(v\right)$ across all v's), where the subscript j=1,…,V is the rank and v as well as $v_{\left(j\right)}$ still denote the original vertex label, which maps back to a specific location in the brain map.
3. In the sorted list of brain–phenotype associations, we identify the $T\left(v_{\left(j\right)}\right)$'s where $v_{\left(j\right)}\in N$ (see horizontal lines in Figure 2b).
4. Initialize a running sum metric for network N at 0 (${\mathrm{RS}}_{\left(0\right)}=0$). Begin walking down the list for j=1,…,V (i.e., starting with $T\left(v_{\left(1\right)}\right)$), the strongest positive association, and ending with $T\left(v_{\left(V\right)}\right)$, the strongest negative association. Increase or decrease the running sum as follows (and as illustrated in Figure 2c):
(6) $${\mathrm{RS}}_{\left(j\right)}=\left\{\begin{array}{ll} {\mathrm{RS}}_{\left(j-1\right)}+\frac{|T\left(v_{\left(j\right)}\right)|}{\sum_{v\in N}|T\left(v\right)|} & \text{if}\;v_{\left(j\right)}\in N\\ {\mathrm{RS}}_{\left(j-1\right)}-\frac{1}{\#N^{c}} & \text{if}\;v_{\left(j\right)}\in N^{c} \end{array}\right.$$
  where the cardinality of the set $N^{c}$ is denoted by $\#N^{c}$ (i.e., the number of locations outside N). Although we focus on the above definition of the running sum metric in this article, we note that researchers interested in conducting a two‐sided test (i.e., testing over‐enrichment *or* under‐enrichment), Equation (6) may be modified so that the increment of increase is $\frac{1}{\#N}$ for $v_{\left(j\right)}\in N$ and decrease it by $\frac{1}{\#N^{c}}$ for $v_{\left(j\right)}\in N^{c}$. That is, for a two‐sided test, the running sum would increase (decrease) by increments proportional to the number of vertices inside (outside) the network, instead of the increment of increase being weighted by the magnitude of $T\left(v_{\left(j\right)}\right)$ for each $v_{\left(j\right)}\in N$.
5. Compute the enrichment score (ES) for network N as the maximum deviation from zero that RS takes on
(7) $${\mathrm{ES}}^{\left(\mathrm{obs}\right)}=\max_{j=1,\ldots ,V}|{\mathrm{RS}}_{\left(j\right)}|.$$
  Importantly, by increasing the RS by an increment proportional to $|T\left(v_{\left(j\right)}\right)|$ in the previous step, the ES quantifies the extent to which associations are both stronger and of the same sign within the network compared to outside of it.
6. For k=1,…,K permutations (e.g., K=999), permute the phenotype across individuals and quantify brain–phenotype associations $T\left(v\right)$ at each location in the permuted sample. Repeat steps 2–5, denoting the ES from the kth permutation by ${\mathrm{ES}}^{\left(k\right)}$.
7. Estimate a *p*‐value for evaluating enrichment of brain–phenotype associations in network N, by comparing ${\mathrm{ES}}^{\left(\mathrm{obs}\right)}$ to ${\mathrm{ES}}^{\left(k\right)},k=1,\ldots ,K$ (distribution of ${\mathrm{ES}}^{\left(\mathrm{obs}\right)}$ under $H_{0}$):

(8) $$p_{N}=\frac{1+\sum_{k=1}^{K}I_{\left({\mathrm{ES}}^{\left(k\right)}>{\mathrm{ES}}^{\left(\mathrm{obs}\right)}\right)}}{K+1},$$

where the smallest possible p‐value is $1/\left(K+1\right)$ (Phipson & Smyth, 2010).

While the steps above return a *p*‐value for a pre‐specified set of network labels N, some steps do not need to be repeated if enrichment is being tested in multiple networks. Specifically, steps 1–2 remain the same across different networks (as does step 6 each time steps 1–2 are reapplied in permuted data). Additionally, we note that the specific approach to permutation (step 6) may depend on the presence of confounders or other nuisance variables, as well as relationships among covariates. This topic has been explored in previous literature, and we refer readers to Winkler et al. (2014)'s work.

Open‐source software and detailed documentation on the implementation of NEST is available at https://smweinst.github.io/nest-method/.

### 2.3 Application in data from the Philadelphia Neurodevelopmental Cohort

To evaluate the performance of NEST, we use data from the Philadelphia Neurodevelopmental Cohort (PNC), a large‐scale study of children, adolescents, and young adults between 8 and 21 years of age (Satterthwaite et al., 2016). A subset of PNC participants underwent multi‐modal neuroimaging at the Hospital of the University of Pennsylvania in the same Siemens TIM Trio 3T scanner under the same protocols, which are detailed in Satterthwaite et al. (2014) and summarized in Appendix A. Utilizing the same data from our earlier work (Weinstein et al., 2021), data were processed using FreeSurfer version 5.3 and the fsaverage5 atlas to reconstruct images on the cortical surface, consisting of 10,242 vertices per hemisphere (Fischl, 2012). After removing the medial wall (i.e., subtracting 888 and 881 vertices from the left and right hemispheres, respectively), V=18,715 vertices remain across both hemispheres for each person. While the medial wall is excluded in our models of brain–phenotype associations, we add these vertices back for the purpose of visualizations using the fsbrain package in R (Schäfer & Ecker, 2020).

In this study, we focus on network enrichment of age and sex effects on brain structure (cortical thickness in mm) for N=911 participants and functional activation during the n‐back task (percent change between the 0‐ and 2‐back sequences) for N=1,018 participants, after applying similar exclusion criteria as in our previous work (Weinstein et al., 2021). In simulation studies and data analyses (Sections 2.3.2 and 2.3.3), we apply NEST to evaluate enrichment of brain–phenotype associations in the seven functional networks described by Yeo et al. (2011). We anticipate that age and sex effects may be especially enriched within the dorsal attention, ventral attention, frontoparietal, and default networks, as increased connectivity within and between these networks play an important role in the maturation of cognitive abilities from childhood to adolescence (Keller et al., 2022; Sydnor et al., 2021). Age‐related differences in brain activity during the n‐back task are often believed to localize within these networks given their involvement in higher‐order functions (Satterthwaite et al., 2013). Applying NEST in analyses of brain–phenotype associations within these networks in a neurodevelopmental cohort may indeed corroborate past findings, while invoking more rigorous methodology.

#### 2.3.1 Quantification of brain–phenotype associations

In this section, we describe our approach to quantifying local linear and nonlinear brain–phenotype associations in the PNC data. To flexibly model brain image measurements as a function of non‐imaging phenotypes, we fit a generalized additive model (GAM) at each vertex. We then use a multivariate Wald statistic to jointly capture linear and nonlinear brain–phenotype associations from each GAM.

Let $x\left(v\right)=\left(x_{1}\left(v\right)\;x_{2}\left(v\right)\;\ldots x_{N}\left(v\right)\right)$ denote measurements of the brain (across participants) at a given location v. We consider the following model for each v and for each type of brain measurement (cortical thickness or n‐back activation):

(9) $$\begin{array}{l} x\left(v\right)=\alpha_{0}^{\left(v\right)}+\alpha_{1}^{\left(v\right)}I_{\left(\mathrm{sex}=\text{female}\right)}+f_{1}^{\left(v\right)}\left(\mathrm{age}\right)+f_{2}^{\left(v\right)}\left(\mathrm{age}\times I_{\left(\mathrm{sex}=\text{female}\right)}\right)\\ \;+\;\varepsilon^{\left(v\right)}\;\text{for}\;v=1,\ldots ,18,715, \end{array}$$

where the superscripts $\left(v\right)$ indicate location (vertex)‐specific components of the model, and 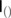 denotes an indicator function. In the model above, $\alpha_{0}^{\left(v\right)}$ and $\alpha_{1}^{\left(v\right)}$ are linear parameters for mean and sex (female) effects, respectively, for each v. The smoothing functions, $f_{1}^{\left(v\right)}\left(\right)$ and $f_{2}^{\left(v\right)}\left(\right)$, are fixed degrees of freedom regression natural cubic splines with $K_{1}$ and $K_{2}$ knots, respectively. We estimate these functions using the mgcv package in R (Wood & Wood, 2015).

The use of these smooth functions means that we estimate multiple different parameters for each effect of interest. However, to spatially map brain–phenotype associations, we would like to collapse information across these parameters into a single vertex‐level statistic, $T\left(v\right)$. To combine information across all parameters associated with each effect of interest in Equation (9), we use a multivariate Wald statistic. Since the Wald statistic is expressed as a quadratic form and will always be positive, we assign a sign of each Wald statistic based on coefficient estimates from multiple linear regression models, which would reflect the overall trend for nonlinear associations. Details on this statistic are outlined in Appendix B.

#### 2.3.2 Simulation studies

We use a resampling procedure to conduct simulation studies using real data from the PNC. Across 1000 simulations, we consider scenarios involving sub‐samples of different sizes ($N_{\mathrm{sub}}=50,100,200,$ or 300) and evaluate whether each brain–phenotype association of interest is enriched within each of the seven functional networks delineated by Yeo et al. (2011). We conduct these simulations in both a simulated null setting (for estimating type I error) and an observed data setting (for estimating power).

##### Type I error simulations

We first evaluate the type I error rates of NEST and two existing approaches: the spin test (Alexander‐Bloch et al., 2018) and FastGSEA (Korotkevich et al., 2016; Park et al., 2018). For a given brain–phenotype association (e.g., cortical thickness and age), we randomly permute the phenotype across participants in the full sample, then randomly select a subset of $N_{\mathrm{sub}}$ (50,100,200, or 300). By permuting the data before subsetting, we force $H_{0}$ to be true, since brain–phenotype associations have been destroyed at all locations, and thus there should be no enrichment. Even in this null setting, we nevertheless anticipate that maps of brain–phenotype associations would still exhibit some degree of spatial smoothness. We expect this may be a problem for methods whose null distributions involve various forms of spatial randomization or permutation. Examples of null brain–phenotype association maps from a single sub‐sample from our simulations are presented in Appendix C (Figure C.1).

**FIGURE 3:**
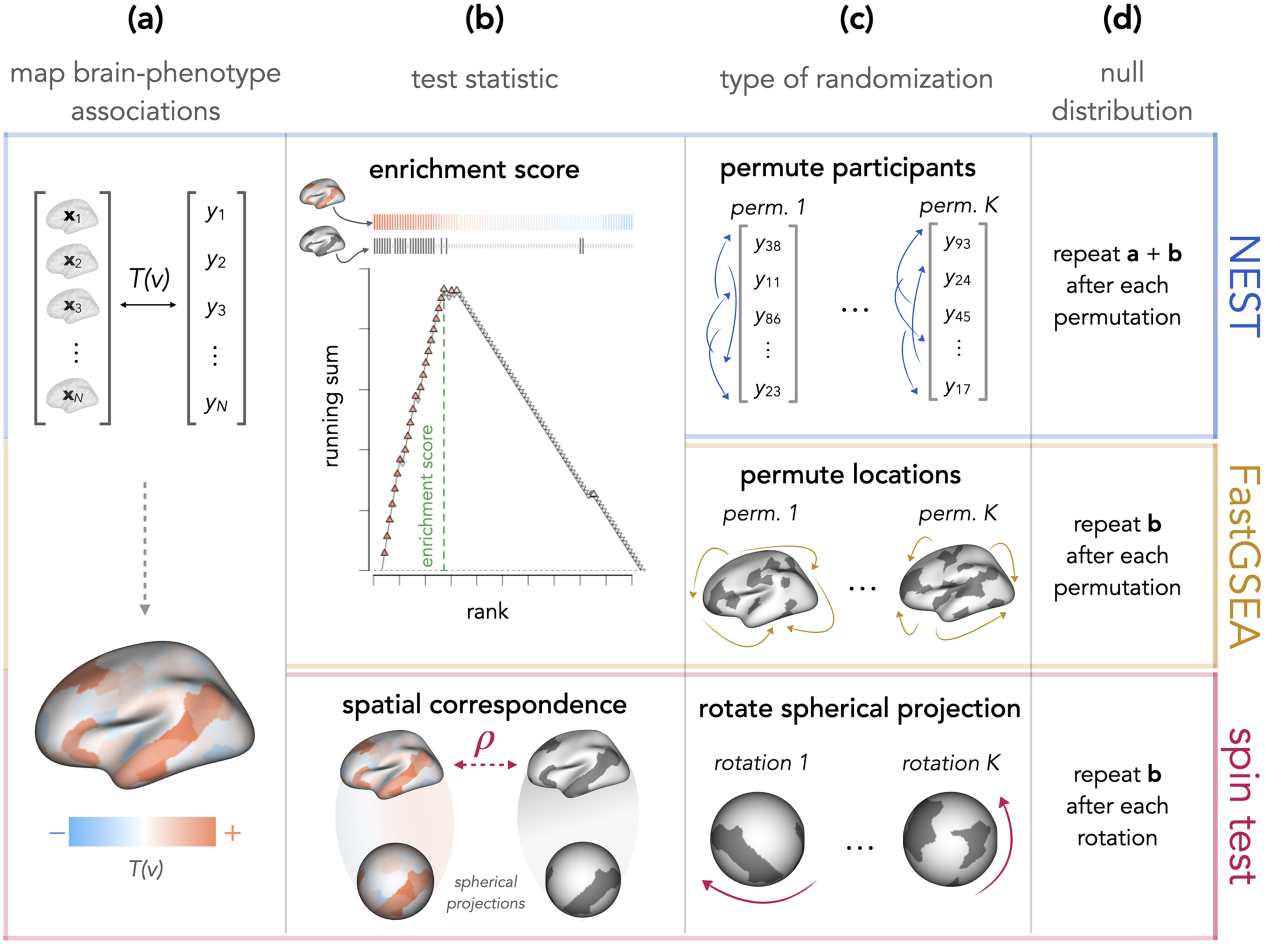
Comparison of our proposed method, NEST, to the spin test (Alexander‐Bloch et al., 2018) and FastGSEA (Korotkevich et al., 2016; Park et al., 2018), which have been used to evaluate network enrichment in previous neuroimaging studies. All three methods begin with (a), estimating brain–phenotype associations at each location. In (b), both NEST (which is based on Subramanian et al.'s (2005) GSEA) and FastGSEA estimate an enrichment score (ES), illustrated in detail in Figure 2. For the spin test, the test statistic is a measure of spatial correspondence between the map of brain phenotype associations and the network partition map. In (c), we illustrate the type of randomization each method uses in order to generate a null distribution. In NEST, a null distribution is formed by ES's computed after permuting participants (also see Figure 2), whereas FastGSEA permutes spatial units (e.g., vertices or parcels). In the spin test, a spherical projection of the network partition map is randomly rotated before recomputing the spatial correspondence measure forming the null distribution.

Figure 3 provides an overview of the key steps involved in each method. For all three methods, we estimate $T\left(v\right)$, our statistic of brain–phenotype associations at each vertex. For all methods, we also compute our “observed” test statistic under $H_{0}$ using the null association maps just described—that is, the enrichment score for NEST and FastGSEA (upper portion of Figure 3b) or a measure of spatial correspondence (we use Pearson correlation) for the spin test (lower portion of Figure 3b). While the observed test statistic is identical for NEST and FastGSEA (for a given network and participant sample), their null distributions are quite different. In FastGSEA, we repeatedly permute *locations* in the network map before re‐computing each ES to form the null distribution. In NEST, we permute *participants*, obtain an entirely new association map (i.e., a new set of $T\left(v\right)$s), and then re‐compute an ES to form the null distribution (Figure 3c,d).

For the spin test, we compute the “observed” (under $H_{0}$) spatial correspondence using a Pearson correlation to compare the same null map of brain–phenotype associations used for NEST and FastGSEA to a binary network partition map (lower portion of Figure 3b). The null distribution consists of correlations between the “observed” null map (same as for the other two methods) and random rotations of the network map (lower portion of Figure 3c,d).

For each brain–phenotype association, each network, each sub‐sample size ($N_{\mathrm{sub}}$), and each method, we repeat these steps for 1000 simulations, estimating p‐values based on 999 permutations (or rotations, for the spin test) per simulation. Type I error rates in each setting are estimated as the proportion of simulations yielding a p‐value smaller than the nominal α=0.05.

##### Power simulations

As our type I error simulations will show, neither FastGSEA nor the spin test control type I error levels in the context of this realistic simulation design. Therefore, we do not consider these methods in our power simulations or in subsequent analyses.

For NEST, we estimate power by applying the method within 1000 sub‐samples of each $N_{\mathrm{sub}}$ (from un‐permuted data). We estimate power as the rate of rejecting the null hypothesis at the α=0.05 level. (Note that we refer to this quantity as “power” for simplicity; however, in settings where the null hypothesis is true, it should resemble type I error).

We also consider the extent to which NEST is robust to the resolution of cortical surface data. After down‐sampling participant‐level brain maps to V=1000, 500, or 200 parcels Schaefer et al. (2018), we repeat our simulations involving enrichment tests of age effects on both cortical thickness and n‐back. We again repeat 1000 simulations per modality, sample size, and Yeo et al. (2011) seven networks. We will present these results in Appendix C.

Lastly, we evaluate NEST's computational efficiency (using our R implementation) when spatial resolution and sample size vary. We also report computation times when linear regression coefficients are used to define $T\left(v\right)$, instead of the GAM‐based multivariate Wald statistic, which may be more computationally intensive. Means and standard deviations (in seconds) are reported across 2000 simulations for each setting (1000 each for cortical thickness and n‐back), since we do not expect NEST's computation time to differ by modality. A summary will be presented in Appendix D.

#### 2.3.3 Data analysis

Following our simulation studies, we apply NEST to evaluate the same brain–phenotype associations using data from the full PNC sample. Again, we quantify associations at the vertex‐level for cortical thickness and n‐back with age, sex, and the interaction between age and sex using the multivariate Wald statistic described in Section 2.3.1, and we test enrichment of associations within each of Yeo et al.'s (2011) seven functional networks. For each test, p‐values are estimated based on K=4,999 permutations.

We report unadjusted p‐values for all seven networks and indicate which remain statistically significant after controlling the false discovery rate (FDR) at q<0.05. In our adjustment for multiple comparisons, we conservatively account for testing across both modalities, three brain–phenotype associations, and seven networks, for a total of 42 tests.

As a post hoc analysis, we also apply NEST to evaluate enrichment of brain–phenotype associations within Yeo et al.'s (2011) 17‐network partition. Given the exploratory nature of these analyses, we do not adjust for multiple comparisons when reporting these results.

## 3 RESULTS

### 3.1 Simulation results

**FIGURE 4:**
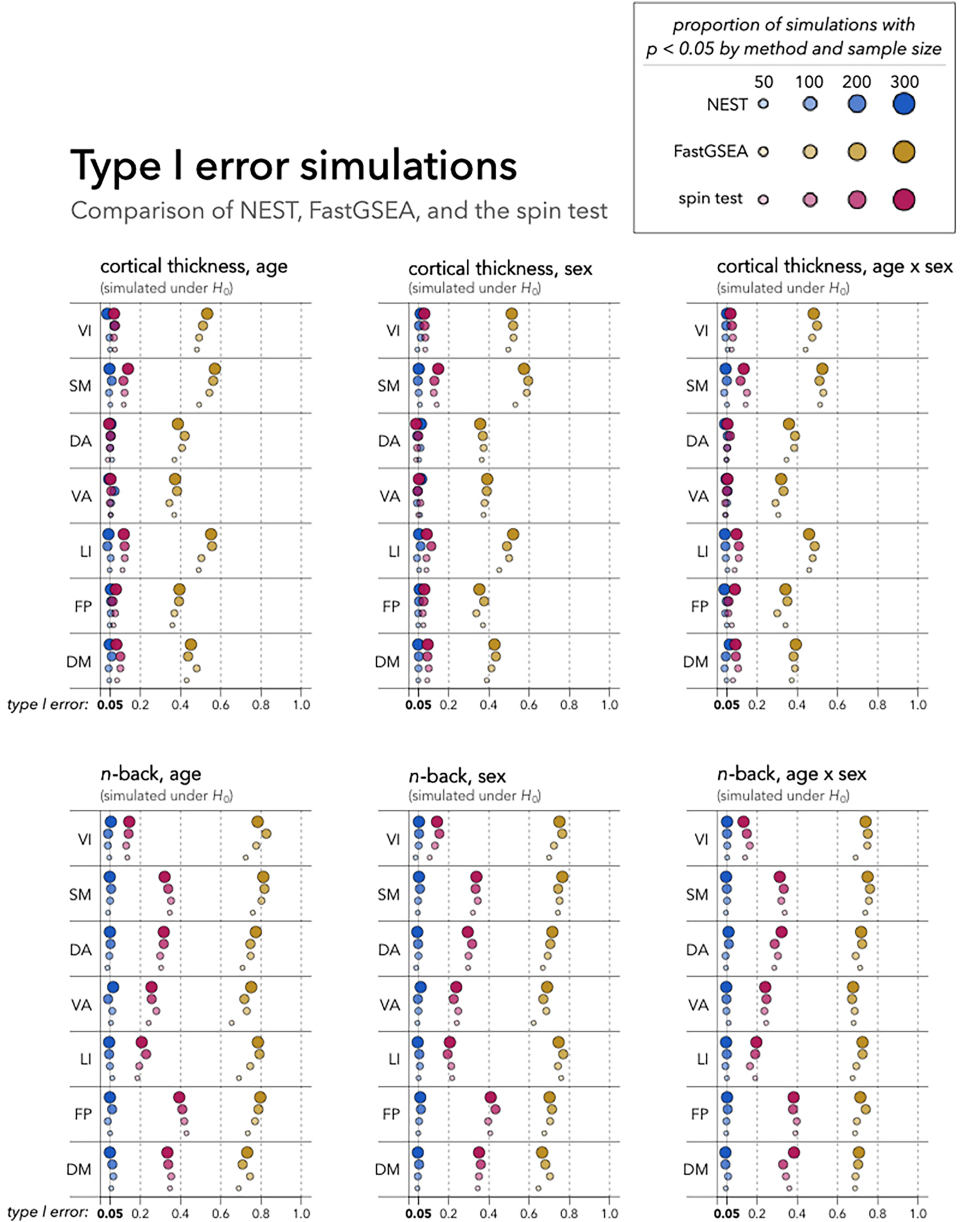
Comparison of type I error levels of our proposed method (NEST), the spin test (Alexander‐Bloch et al., 2018), and FastGSEA (Korotkevich et al., 2016; Park et al., 2018) based on 1000 random sub‐samples of different sizes (50, 100, 200, or 300). In each sub‐sample, p‐values are obtained from each method (based on 999 permutations for NEST and FastGSEA, or 999 random rotations for the spin test). Type I error estimates with 95% binomial confidence intervals are provided in Table C.1 of Appendix C. Our results suggest that NEST controls type I errors at the nominal level (α=0.05) in null simulations involving all six brain–phenotype associations considered, all networks, and all sample sizes ($N_{\mathrm{sub}}$). In contrast, neither the spin test nor FastGSEA control type I error levels, suggesting these methods may not be reliable in tests of network enrichment. We speculate that this may be due to inherent spatial smoothness of null brain–phenotype association maps, even in the absence of genuine network enrichment. Examples of simulated null association maps involving the n‐back task are shown in Figure C.1 of Appendix C. DA, dorsal attention; DM, default mode; FP, frontoparietal; LI, limbic; SM, somatomotor; VA, ventral attention; VI, visual.

As described in Section 2.3.2, our type I error simulation studies evaluate the rate at which each method rejects its null hypothesis in settings where our proposed null hypothesis (as defined in Section 2.1), is true. Results are presented in Figure 4, with different color points used for each method (blue for NEST, yellow for FastGSEA, and pink for spin) and different shades and sizes used to distinguish between sample sizes, where the darkest/largest dot corresponds to the largest sub‐sample size ($N_{\mathrm{sub}}=300$) and the lightest/smallest dot corresponds to the smallest sample size ($N_{\mathrm{sub}}=50$). We also report these results with 95% confidence intervals in Table C.1 of Appendix C.

NEST successfully controls type I error rates at the nominal level (α=0.05) across all simulated null brain–phenotype associations, all sample sizes, and all seven networks. In contrast, neither the spin test nor FastGSEA controls type I error levels. For the spin test, type I error estimates range from 10.5% to as high as 43.2% for null simulations involving the n‐back task and from 3.7% to 14.9% for those involving cortical thickness. Although the spin test does control type I error levels in certain situations (e.g., simulated nulls involving cortical thickness‐age, sex, and age × sex associations in the ventral attention network), more often, we reject the null at a rate that exceeds the nominal level. FastGSEA also fails to control false positive rates, with type I error estimates ranging from 29.1%, at best, to 82.6%, at worst.

Interestingly, both the spin test and FastGSEA appear to have even higher type I error levels in null simulations involving the n‐back task compared to those involving cortical thickness. We speculate that this may be due to the extent of spatial autocorrelation found in null maps involving the n‐back task compared to those involving cortical thickness. To explore this possibility, Appendix C displays an example simulated null map from each type I error simulation setting (i.e., all six brain–phenotype association combinations in both observed and null settings). From a visual inspection of these maps, it appears that there is a greater degree of spatial smoothness in maps involving the n‐back task compared to those involving cortical thickness. The extent of spatial smoothness may therefore contribute to the extent of type I error inflation for the spin test and FastGSEA, as both these methods make strong assumptions about the spatial structure of the data.

**FIGURE 5:**
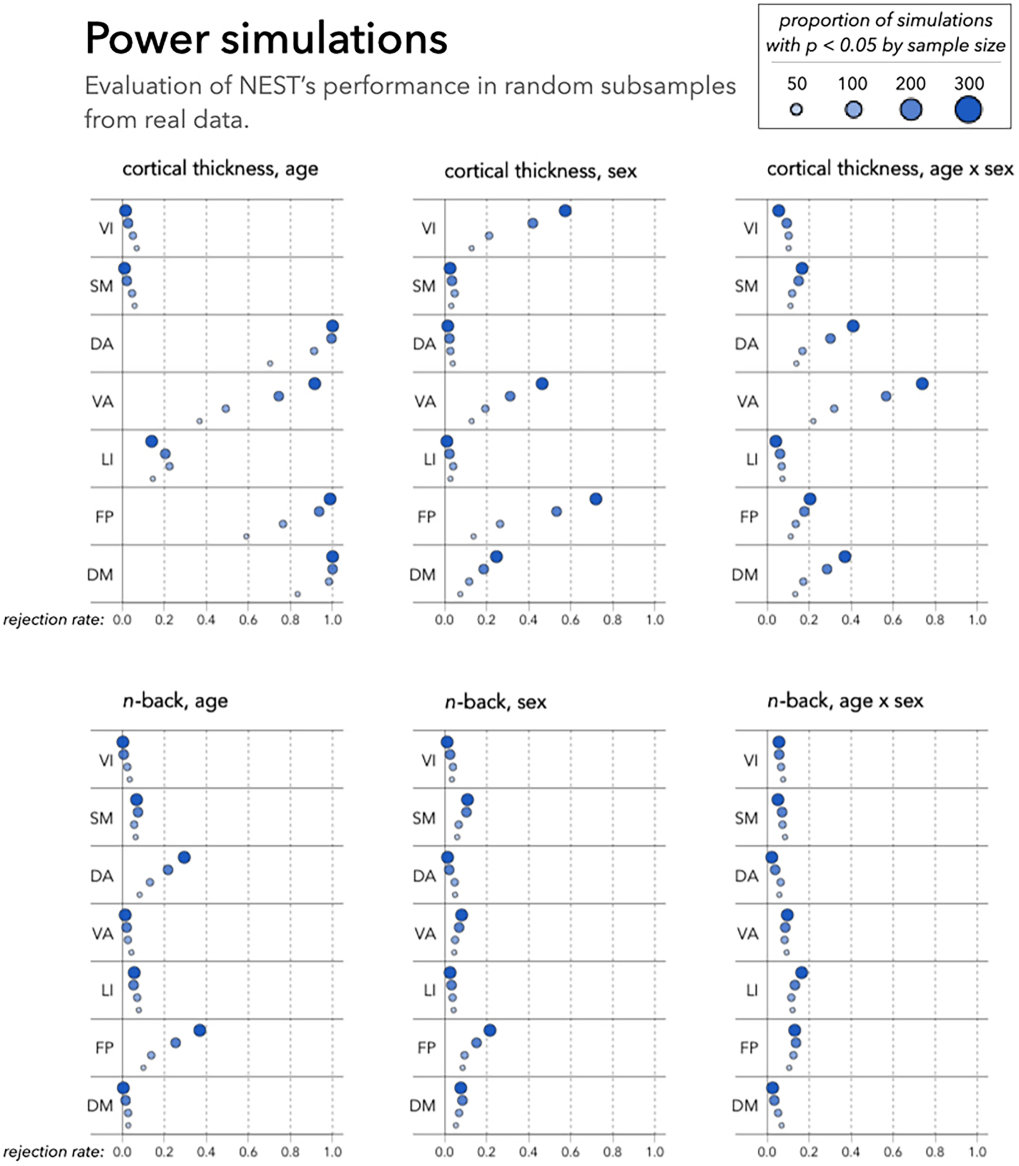
Power of NEST based on data‐driven simulation studies. We randomly select subsamples of different sizes (50, 100, 200, or 300) from the Philadelphia Neurodevelopmental Cohort and apply NEST to test enrichment of age, sex, and age × sex effects on cortical thickness (top row) and n‐back activation (bottom row) in each of the seven networks delineated by Yeo et al. (2011). Given results from our type I error simulations (Figure 4), assessing power of FastGSEA or the spin test would not be meaningful. DA, dorsal attention; DM, default mode; FP, frontoparietal; LI, limbic; SM, somatomotor; VA, ventral attention; VI, visual.

Given the results of our type I error simulations, we exclude both FastGSEA and the spin test from subsequent results, as these methods' proneness to inflated false positive findings calls into question their interpretation. In Figure 5, we present the statistical power of NEST after repeatedly applying this method in each randomly selected sub‐sample from the full observed (i.e., not permuted) PNC sample, with 95% confidence intervals reported in Table C.2 of Appendix C. As noted earlier, in the context of our power simulations, the null hypothesis may be either true or false, but for simplicity, we refer to the rate of rejecting $H_{0}$ as power. For cortical thickness‐age effects, NEST's power is highest in the default, frontoparietal, and dorsal and ventral attention networks. In the default network, we estimate power at 100% when $N_{\mathrm{sub}}$ = 200 or 300, 98.3% when $N_{\mathrm{sub}}=100$, and 83.4% when $N_{\mathrm{sub}}=50$. In the dorsal attention network, power is estimated at 100% when $N_{\mathrm{sub}}=300$, 99.5% when $N_{\mathrm{sub}}=200$, 91.2% when $N_{\mathrm{sub}}=100$, and 70.3% when $N_{\mathrm{sub}}=50$. In the frontoparietal and ventral attention networks, the reduction in power due to decreased sample size is more notable. For the frontoparietal network, the estimated power exceeds 90% when $N_{\mathrm{sub}}\geq 200$, but declines to 76.4% when $N_{\mathrm{sub}}=100$ and to 59.0% when $N_{\mathrm{sub}}=50$. For the ventral attention network, power exceeds 90% when $N_{\mathrm{sub}}=300$ but declines to 74.4% when $N_{\mathrm{sub}}=200$, to 49.2% when $N_{\mathrm{sub}}=100$, and to 36.7% when $N_{\mathrm{sub}}=50$.

For enrichment of associations involving the n‐back task, it appears that larger sample sizes (beyond those considered in these simulations) may be required to detect enrichment of associations where we would expect them (for instance, age effects in the frontoparietal network). In Section 4, we discuss potential future directions that may help improve NEST's power in settings with smaller sample sizes.

#### 3.1.1 Statistical power and computational efficiency at different resolutions

In Appendix C, we report NEST's power when participant‐level cortical surface data at lower resolutions (V = 1000, 500, or 200 across both hemispheres) are considered in enrichment tests involving age effects on cortical thickness and n‐back. For network enrichment of cortical thickness‐age associations (Table C.3a), simulation settings using coarser parcellations (i.e., smaller V) tend to have lower power than those with finer parcellations (i.e., larger V), and power tends to be especially low when both V and $N_{\mathrm{sub}}$ are smaller. However, there may be settings where using a coarser parcellation may improve power (e.g., in the ventral attention network across all sample sizes, power is highest when V=500), and this warrants additional consideration in future extensions of this work. For network enrichment of n‐back‐age associations (Table C.3b), similar to the results of our simulations involving vertex‐level data, power is relatively low across all sample sizes and resolutions, suggesting a need for either larger sample sizes or other adaptions of the method to improve power (see Section 4). In Appendix D, we summarize NEST's computation time (in seconds) from our simulation studies.

### 3.2 Data analysis results

**FIGURE 6:**
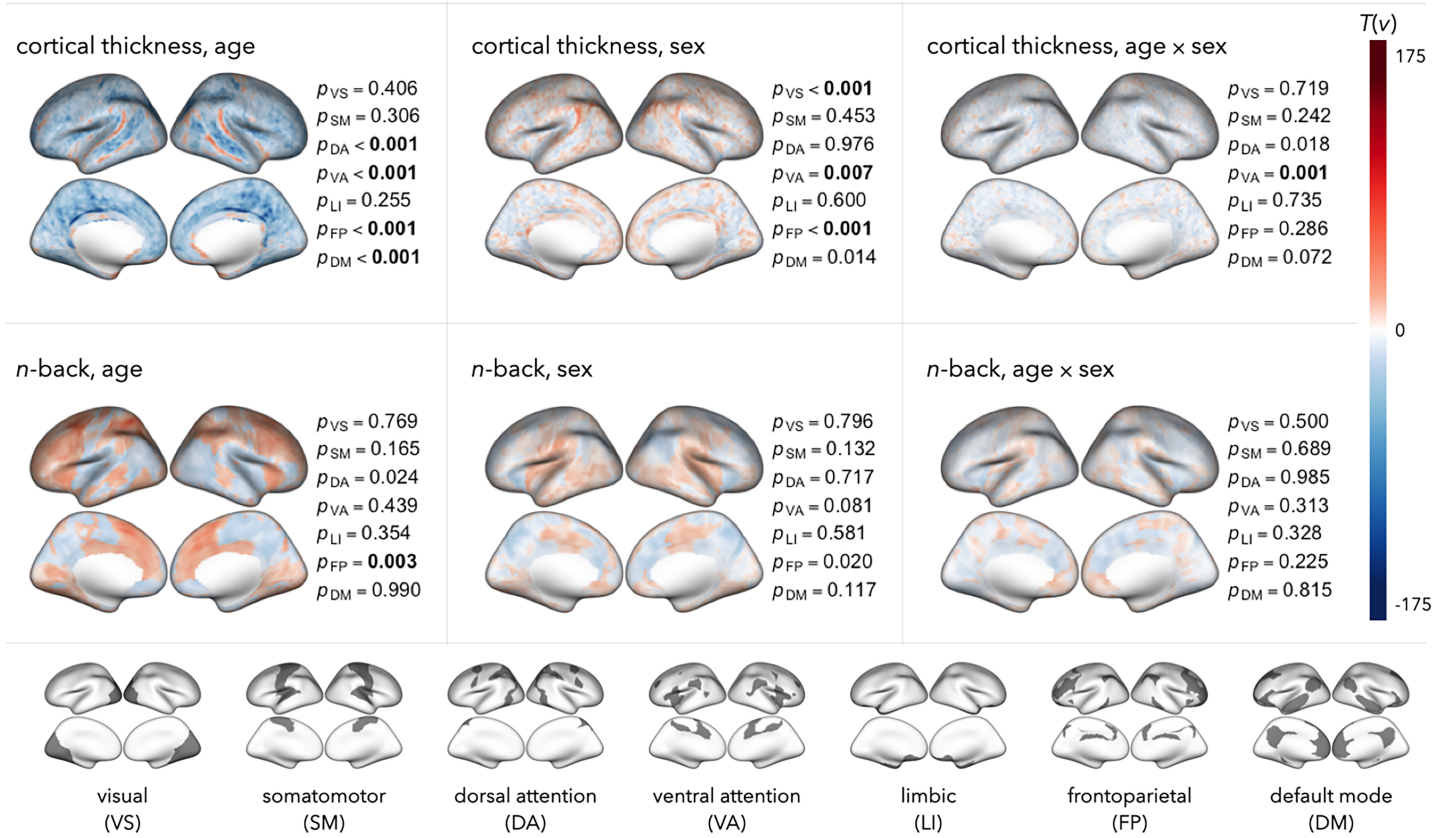
Results from application of NEST to evaluate network enrichment of associations between cortical thickness or n‐back with age, sex, or age × sex interactions. For each setting, we map association statistics (signed multivariate Wald statistic, as described in Section 2.3.1 and Appendix B) across the cortical surface. Unadjusted p‐values based on K = 4999 permutations are reported for each of Yeo et al.'s (2011) seven network partitions (displayed at the bottom), with bold text indicating results that remain statistically significant after controlling the false discovery rate (FDR) at q<0.05 across both brain measurements (cortical thickness, n‐back), all three phenotypes (age, sex, age × sex), and all seven networks. Interpretation of age effects: shades of red (blue) indicate locations where the brain measurement is estimated to increase (decrease) nonlinearly with age, including nonlinear age effects that may differ by sex. Interpretation of sex effects: shades of red (blue) indicate locations where the brain measurement is estimated to be higher (lower) for females versus males, including sex differences that may vary nonlinearly with age. Interpretation of age × sex effects: shades of red (blue) indicate locations where nonlinear age effects were estimated to be higher (lower) for females versus males.

In Figure 6, we present results from applying NEST in the full PNC cohort to evaluate the same brain–phenotype associations and networks as in our simulation studies. Unadjusted p‐values are reported, with bolded text in Figure 6 indicating results that remain statistically significant after FDR control.

To guide in the interpretation of Figure 6, we note that for maps involving associations with age, positive values of $T\left(v\right)$ (shown in shades of red) indicate areas where either cortical thickness or n‐back activation is typically found to increase (nonlinearly) with age, while negative values (shades of blue) are where we estimate the brain measurement to decrease with age. For cortical thickness‐age associations, we find significant enrichment of nonlinear age effects in the default, frontoparietal, ventral attention, and dorsal attention networks. For n‐back‐age associations, we find significant enrichment in the frontoparietal network.

For interpreting spatial maps of associations with sex, positive values of $T\left(v\right)$ indicate locations where we find either cortical thickness or n‐back activation to be higher for females compared with males, while negative values of $T\left(v\right)$ indicate locations where the brain measurement is found to be lower for females. After FDR correction, we find significant enrichment of sex differences in cortical thickness for the visual, ventral attention, frontoparietal, and default mode networks. We find no significant enrichment of sex effects in n‐back activation in any networks.

For network enrichment of age × sex interactions, positive values of $T\left(v\right)$ are interpreted as regions where nonlinear age effects tended to be higher among females compared to males, while negative values indicate locations where nonlinear age effects were generally found to be lower among females. We find significantly enriched age × sex effects on cortical thickness within the dorsal attention, ventral attention, and default mode network. We do not find significant enrichment of these effects on n‐back in any of the seven networks.

In Table C.4 (Appendix C), we report p‐values from our post hoc analyses, after applying NEST to evaluate enrichment of the same brain–phenotype associations within sub‐networks of our primary analysis. While these analyses are exploratory (and are therefore not adjusted for multiple testing), the results within the 17‐network partition seem to corroborate (and add specificity to) those from the 7‐network analysis.

## 4 DISCUSSION

Interpreting brain–phenotype associations within the context of functional network maps is a common practice in neuroimaging studies. However, existing methods for testing network specificity have fallen short by relying on subjective interpretations, strong statistical assumptions, or ignoring complex distributional features of neuroimaging data. In this article, we attempt to address this gap by extending GSEA (Subramanian et al., 2005), a method that has been used for nearly two decades in genetics research, to the neuroimaging setting.

Translating GSEA to the setting of studying individual differences in brain anatomy and function, NEST begins by estimating local (e.g., vertex‐level) brain–phenotype associations (Figure 2a). We then compute an “enrichment score” (ES) for a network of interest, which quantifies the extent to which locations where the brain measurement is strongly associated with the phenotype tend to occur inside versus outside the network of interest (Figure 2c–e). We then construct a null distribution by re‐computing the ES after repeatedly permuting the original data across participants and re‐computing local brain–phenotype associations (Figure 2b). We estimate a p‐value by comparing the observed ES to this permutation‐based null distribution (Figure 2e).

We applied NEST to structural and functional brain surface maps from the Philadelphia Neurodevelopmental Cohort (Satterthwaite et al., 2014). Our simulation studies illustrate that NEST controls type I error levels, whereas two previous methods do not (Figure 4). Our results from power simulations and full‐sample data analyses suggest that NEST tends to perform as expected; however, with its current implementation, larger sample sizes may be needed to achieve adequate power. In future work, it would be interesting to explore whether we can improve power when large sample sizes are not available or feasible to collect. Indeed, our simulation studies revealed a noticeable reduction in power in smaller samples (Figure 5), especially for enrichment tests involving associations with the n‐back task. Additionally, while in this article, we quantified local brain–phenotype associations ($T\left(v\right)$) separately at each vertex, in future work, we hope to explore whether we can improve power by leveraging spatial information in the construction of $T\left(v\right)$—for instance, through cluster enhancement (Park & Fiecas, 2022) or Bayesian spatial modeling (Spencer et al., 2022).

Until recently, researchers often relied on ad hoc or subjective methods to make claims about the specificity of brain–phenotype associations to functional networks. For instance, visualizing a brain map of association statistics next to a network partition map, researchers would comment on the appearance of overlap in brain regions with especially large statistics with maps of network partitions. Another approach would be to note the percentage of vertices or voxels with strong associations overlapping with networks of interest. While the spin test (Alexander‐Bloch et al., 2018) has become increasingly popular, it is questionable whether the null hypothesis it tests—that there is no spatial alignment between two maps—is an appropriate translation of questions it is often used to answer. Combined with previous critiques of the spin test (e.g., Pang et al., 2023), results from our simulation studies indeed call its reliability into question. Specifically, we found inflated type I error rates in simulated null settings where enrichment was not present. Thus, significant spatial alignment (as one might interpret a small p‐value from the spin test) does not necessarily correspond to spatial enrichment, as defined in this article. This is likely due to greater smoothness found within networks (versus between networks), which is present even in null association maps (e.g., Figure C.1 in Appendix C). Similar properties would also underlie FastGSEA's severe type I error inflation. NEST, on the other hand, does not rely on such assumptions for valid inference. Instead, NEST leverages participant‐level data in the generation of a null distribution, and participants (rather than spatial locations) are the unit of randomization. In practice, methods for testing enrichment without participant‐level data may still be needed in some studies, and future methods might consider alternative approaches to accomplish this without relying on strong assumptions.

NEST has several limitations, opening up several directions for future research. Firstly, our proposed definition of network enrichment assumes sign‐consistency of brain–phenotype associations within an enriched network. In future work, it may be useful to consider more flexible definitions of network enrichment to encompass sign‐inconsistency. Also, since NEST requires that users pre‐define the region(s) in which to test enrichment, in its current form, it is likely most useful in confirmatory research. However, aspects of the method may be incorporated in exploratory analyses and hypothesis generation—for example, for a given brain–phenotype association map, one could compare enrichment scores associated with a variety of alternate network partitions.

Relatedly, we have yet to compare NEST's performance when networks of varying sizes (relative to the resolution of the image) are of interest. Importantly, researchers using GSEA have proposed certain rules of thumb regarding minimum and maximum sizes of gene sets, suggesting that sensitivity and interpretability may be compromised when gene sets are too large or small (Maleki et al., 2020; Mooney & Wilmot, 2015; Simillion et al., 2017). However, the proposed cutoffs have been somewhat arbitrary and may not generalize to studies of different gene–phenotype relationships. Therefore, our current recommendation is for researchers using NEST to rely on subject‐area expertise to determine which networks to test, rather than arbitrary cutoffs for network size. In future work, we hope to evaluate whether it is either useful or possible to provide more general guidelines on this topic.

While assessing network specificity of brain–phenotype associations is quite common in neuroimaging studies, existing approaches are severely limited by either subjectivity or reliance on strong assumptions. NEST is the first method, to our knowledge, that circumvents these limitations.

## AUTHOR CONTRIBUTIONS

**Sarah M. Weinstein:** Conceptualization, methodology, software, writing (original draft), reviewing, and editing. **Simon N. Vandekar:** Supervision, methodology, writing, reviewing, and editing. **Bin Li:** Software development, methodology, reviewing, and editing. **Aaron F. Alexander‐Bloch:** Methodology, software, writing, reviewing, and editing. **Armin Raznahan:** Methodology, writing, reviewing, and editing. **Mingyao Li:** Methodology, writing, reviewing, and editing. **Raquel E. Gur:** Data collection, data processing, writing, reviewing, and editing. **Ruben C. Gur:** Data collection, data processing, writing, reviewing, and editing. **David R. Roalf:** Data collection, writing, reviewing, and editing. **Min Tae M. Park:** Conceptualization, writing, reviewing, and editing. **Mallar Chakravarty:** Conceptualization, writing, reviewing, and editing. **Erica B. Baller:** Data processing, writing, reviewing, and editing. **Kristin A. Linn:** Supervision, methodology, writing, reviewing, and editing. **Theodore D. Satterthwaite:** Supervision, conceptualization, data collection, data processing, writing, reviewing, and editing. **Russell T. Shinohara:** Supervision, conceptualization, methodology, writing, reviewing, and editing.

## CONFLICT OF INTEREST STATEMENT

Russell T. Shinohara receives consulting income from Octave Bioscience and compensation for reviewership duties from the American Medical Association. Aaron Alexander‐Bloch receives consulting income from Octave Bioscience and holds equity and serves on the board of directors of Centile Biosciences. Mingyao Li receives research funding from Biogen Inc. that is unrelated to the current manuscript.

## Supporting information


**Data S1:** Supporting information.

## Data Availability

Neuroimaging data were acquired as part of the Philadelphia Neurodevelopmental Cohort (PNC). PNC data are publicly available in raw format at https://www.ncbi.nlm.nih.gov/projects/gap/cgi-bin/study.cgi?study_id=phs000607.v3.p2. Software and documentation are available at https://smweinst.github.io/nest-method/.
