# Supplementary material for: Network enrichment significance testing in brain–phenotype association studies
Source: Hum Brain Mapp. 2024 Jun 15;45(8):e26714. doi: 10.1002/hbm.26714 (PMC11179683; doi:10.1002/hbm.26714)
Supplement: Supplementary file 1 — Data S1: Supporting information. [file HBM-45-e26714-s001.pdf]

Appendix

Appendix A Data collection and pre-processing

In this study, we use data from the Philadelphia Neurodevelopmental Cohort (PNC), a large-scale research study of children, adolescents, and young adults led by researchers at the University of Pennsylvania and Children's Hospital of Philadelphia (Satterthwaite et al. 2014; Satterthwaite et al. 2016). Participants ($N = 1,601$) underwent MRI scans in a Siemens TIM Trio 3 tesla machine with a 32-channel head coil. Imaging sequences and parameters are detailed in previous work (Satterthwaite et al. 2014; Satterthwaite et al. 2016). After exclusion criteria (see Weinstein et al. (2021)), we include $N = 911$ participants in our analysis of cortical thickness data and $N = 1,018$ participants in our analysis of n-back activation.

Structural MRI scanning protocols involved a magnetization-prepared, rapid-acquisition gradient echo (MPRAGE) T1-weighted image with $0.9 \times 0.9 \times 1.0$ mm voxel resolution, and image quality was rated by three experienced analysts (Rosen et al. 2018). Using FreeSurfer (version 5.3), we converted T1-weighted images to cortical surface data (including template registration, intensity normalization, and inflation of cortical surfaces to the fsaverage5 template). We quantified cortical thickness as the minimum distance (in mm) between the pial and white matter surfaces (Dale et al. 1999).

For functional imaging during the n-back task, a single-shot-interleaved multi-slice, gradient-echo, echo planar imaging sequence with voxel resolution of $3 \times 3 \times 3$ mm was used. We used the eXtensible Connectivity Pipeline (XCP) Engine to preprocess the data and mitigate noise induced by head motion (Ciric et al. 2018). During the n-back task, participants viewed geometric stimuli and were instructed to press a button if a present stimulus matched the nth last one they viewed. In this study, we examined n-back activation maps quantifying local percent changes in brain activation between the 2-back and 0-back (i.e., pressing button regardless of relationship between current and previous stimuli) task (Ragland et al. 2002).

Appendix B Metric for quantifying brain-phenotype associations

In this study, we quantify location (v)-specific brain-phenotype associations, $T(v)$, using signed multivariate Wald statistics. The following steps describe how we obtain this metric.

1. We consider the model of brain measurement $\mathbf{x}(v)$ (as a function of phenotypes or covariates) at each image location (v), as presented in presented in Equation (**??**):

   $$x(v) = \alpha_0^{(v)} + \alpha_1^{(v)} \mathrm{I}_{(\text{sex = female})} + f_1^{(v)}(\text{age}) + f_2^{(v)}(\text{age} \times \mathrm{I}_{(\text{sex = female})}) + \epsilon^{(v)} \text{ for } v = 1, \dots, 18,715,$$

   where $f_1^{(v)}()$ allows for nonlinear age effects ($f_1^{(v)}(\text{age}) = \sum_{k=1}^{K_1} \beta_{1k} b_{1k}(\text{age})$), and $f_2^{(v)}()$ allows for nonlinear age effects that may differ by sex ($f_2^{(v)}(\text{age} \times \mathrm{I}_{(\text{sex = female})}) = \sum_{k=1}^{K_2} \beta_{2k} b_{2k}(\text{age} \times \mathrm{I}_{(\text{sex = female})})$) at location v.

2. Let $\hat{\theta}^{(v)}$ denote a vector of all the coefficients estimated in (1) for a given v, including $\hat{\alpha}_0^{(v)}$ (mean), $\hat{\alpha}_1^{(v)}$ (linear age effect), and all the $\hat{\beta}_{1k}^{(v)}$s (nonlinear age effects) and $\hat{\beta}_{2k}^{(v)}$s (nonlinear age $\times$ sex effects). Let $\hat{\Sigma}^{(v)}$ denote the variance/covariance matrix of $\hat{\theta}^{(v)}$. Let Y denote the design matrix for the GAM in (1) and $\hat{\epsilon}^{(v)} = (x(v) - Y\hat{\theta}^{(v)})$ (residuals).

   $$\hat{\Sigma}^{(v)} = \frac{1}{N - P - 1}(Y^T Y)^{-1} \hat{\epsilon}^{(v)T} \hat{\epsilon}^{(v)}$$

   where $P = 2 + K_1 + K_2$ (number of parameters estimated in (1)). Note: the dimension of $\hat{\Sigma}^{(v)}$ is $P \times P$, with each row/column corresponding to a different parameter. In the next step, we use subscripts to denote sub-sets of the vector $\hat{\theta}$ and matrix $\hat{\Sigma}$ corresponding to specific parameters being captured in the Wald statistic.

3. The multivariate Wald statistics for testing the absence of sex, age, and age $\times$ sex effects are defined as follows:

   (a) $W_{\text{sex}}(v) = \hat{\theta}_{\text{sex}}^{(v)T} \hat{\Sigma}_{\text{sex}}^{(v)-1} \hat{\theta}_{\text{sex}}^{(v)}$

   (b) $W_{\text{age}}(v) = \hat{\theta}_{\text{age}}^{(v)T} \hat{\Sigma}_{\text{age}}^{(v)-1} \hat{\theta}_{\text{age}}^{(v)}$

   (c) $W_{\text{age} \times \text{sex}}(v) = \hat{\theta}_{\text{age} \times \text{sex}}^{(v)T} \hat{\Sigma}_{\text{age} \times \text{sex}}^{(v)-1} \hat{\theta}_{\text{age} \times \text{sex}}^{(v)}$

4. While the GAM considered above captures nonlinear associations, we still would like to approximate the overall trend in direction of each association with a (positive/negative) sign. For this, we use coefficient estimates from vertex-level multiple regression models. For age and sex effects, we fit the following model with no interaction term:

   $$x(v) = \gamma_0^{(v)} + \gamma_{\text{sex}}^{(v)} \mathrm{I}_{(\text{sex = female})} + \gamma_{\text{age}}^{(v)} \text{age} + \eta^{(v)}, \tag{1}$$

   so that the signs of the coefficient estimates, $\text{sign}(\hat{\theta}_{\text{sex}}^{(v)})$ and $\text{sign}(\hat{\theta}_{\text{age}}^{(v)})$ reflect both the marginal and interaction effects. To obtain the sign for the age $\times$ sex interaction, we again fit the model in Equation (1) but with an interaction term included (i.e., $+\theta_{\text{age} \times \text{sex}}^{(v)}(\text{age} \times \mathrm{I}_{(\text{sex = female})})$ in Equation (1)).

5. Our association metric for a given brain-phenotype association at location v, $T(v)$, is the product of the multivariate Wald statistic obtained in step (3) and the sign of the corresponding estimated coefficient from the linear model in step (4).

Appendix C Supplementary results

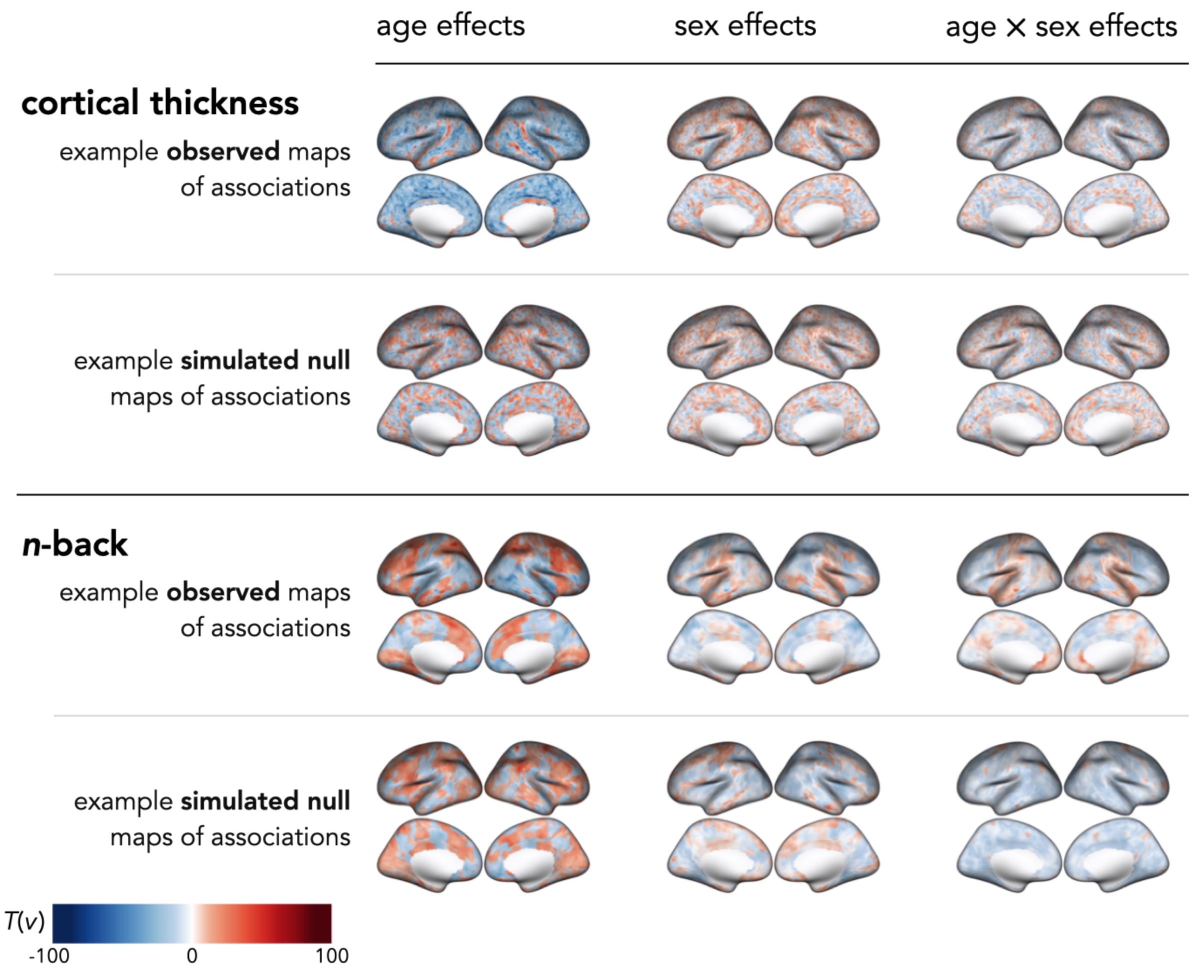

Figure C.1. Example maps of brain-phenotype associations used in data-driven simulation studies for a sub-sample of $N_{sub} = 300$ from the Philadelphia Neurodevelopmental Cohort. Local associations ($T(v)$) for each brain-phenotype combination are calculated using the multivariate Wald statistic described in Section **??** and Appendix B. The observed maps are based on directly calculating associations on the random PNC sub-sample. These correspond to the $T(v)$ maps (from a single simulation) used to estimate an observed enrichment score in the context of our power simulations presented in Figure **??**. The simulated null maps are calculated after first permuting the full PNC sample before subsequently drawing a random sample of N_{sub}=300. Such maps represent "observed" brain-phenotype associations in the context of our type I error simulations presented in Figure **??**.

Table C.1. Type I error rates for tests of network enrichment using NEST, FastGSEA (Korotkevich et al. 2016; Park et al. 2018), and the spin test (Alexander-Bloch et al. 2018) in random subsamples (of different sizes, N_{sub}) from the Philadelphia Neurodevelopmental Cohort. 95% binomial confidence intervals are shown in brackets. These results are also plotted in Figure **??**.

Table C.1(a) Type I error rates for cortical thickness-age associations (simulated under H_0).
Note: these results correspond to the top left panel of Figure **??**.

		$N_{sub} = 50$	$N_{sub} = 100$	$N_{sub} = 200$	$N_{sub} = 300$
Visual	NEST	0.05 [0.04, 0.06]	0.05 [0.03, 0.06]	0.07 [0.06, 0.09]	0.04 [0.03, 0.05]
	FastGSEA	0.48 [0.45, 0.51]	0.49 [0.46, 0.52]	0.51 [0.48, 0.54]	0.53 [0.50, 0.56]
	spin	0.07 [0.06, 0.09]	0.07 [0.05, 0.09]	0.07 [0.06, 0.09]	0.07 [0.05, 0.09]
Somatomotor	NEST	0.05 [0.04, 0.07]	0.04 [0.03, 0.06]	0.06 [0.04, 0.07]	0.05 [0.03, 0.06]
	FastGSEA	0.49 [0.46, 0.52]	0.54 [0.51, 0.57]	0.56 [0.53, 0.59]	0.57 [0.54, 0.60]
	spin	0.12 [0.10, 0.14]	0.12 [0.10, 0.14]	0.12 [0.10, 0.14]	0.14 [0.12, 0.16]
Dorsal Attention	NEST	0.06 [0.05, 0.08]	0.05 [0.04, 0.07]	0.05 [0.04, 0.07]	0.05 [0.04, 0.07]
	FastGSEA	0.37 [0.34, 0.40]	0.41 [0.38, 0.44]	0.42 [0.39, 0.45]	0.39 [0.36, 0.42]
	spin	0.04 [0.03, 0.05]	0.05 [0.04, 0.06]	0.05 [0.04, 0.07]	0.04 [0.03, 0.06]
Ventral Attention	NEST	0.05 [0.04, 0.07]	0.05 [0.04, 0.07]	0.07 [0.06, 0.09]	0.04 [0.03, 0.06]
	FastGSEA	0.37 [0.34, 0.40]	0.34 [0.32, 0.37]	0.38 [0.35, 0.41]	0.37 [0.34, 0.40]
	spin	0.05 [0.04, 0.07]	0.05 [0.04, 0.06]	0.06 [0.04, 0.07]	0.05 [0.04, 0.07]
Limbic	NEST	0.05 [0.04, 0.06]	0.05 [0.04, 0.07]	0.04 [0.03, 0.05]	0.04 [0.03, 0.06]
	FastGSEA	0.49 [0.46, 0.52]	0.50 [0.47, 0.53]	0.56 [0.53, 0.59]	0.55 [0.52, 0.58]
	spin	0.11 [0.09, 0.13]	0.12 [0.10, 0.14]	0.12 [0.10, 0.14]	0.12 [0.10, 0.14]
Frontoparietal	NEST	0.05 [0.04, 0.06]	0.05 [0.04, 0.07]	0.05 [0.04, 0.07]	0.05 [0.04, 0.07]
	FastGSEA	0.36 [0.33, 0.39]	0.37 [0.34, 0.40]	0.39 [0.36, 0.42]	0.39 [0.36, 0.42]
	spin	0.07 [0.05, 0.08]	0.07 [0.06, 0.09]	0.06 [0.05, 0.08]	0.08 [0.06, 0.10]
Default	NEST	0.04 [0.03, 0.06]	0.04 [0.03, 0.06]	0.06 [0.04, 0.07]	0.05 [0.04, 0.06]
	FastGSEA	0.43 [0.40, 0.46]	0.48 [0.45, 0.51]	0.44 [0.41, 0.47]	0.45 [0.42, 0.48]
	spin	0.08 [0.07, 0.10]	0.10 [0.08, 0.12]	0.10 [0.08, 0.12]	0.08 [0.06, 0.10]

Table C.1(b) Type I error rates for cortical thickness-sex associations (simulated under H_0). Note: these results correspond to the top middle panel of Figure ??.

		$N_{sub} = 50$	$N_{sub} = 100$	$N_{sub} = 200$	$N_{sub} = 300$
Visual	NEST	0.04 [0.03, 0.06]	0.06 [0.05, 0.08]	0.05 [0.04, 0.07]	0.06 [0.05, 0.08]
	FastGSEA	0.50 [0.47, 0.53]	0.52 [0.49, 0.55]	0.52 [0.49, 0.55]	0.51 [0.48, 0.54]
	spin	0.08 [0.07, 0.10]	0.08 [0.07, 0.10]	0.08 [0.06, 0.10]	0.08 [0.06, 0.10]
Somatomotor	NEST	0.06 [0.04, 0.07]	0.05 [0.04, 0.07]	0.05 [0.03, 0.06]	0.05 [0.04, 0.07]
	FastGSEA	0.53 [0.50, 0.56]	0.59 [0.56, 0.62]	0.60 [0.56, 0.62]	0.57 [0.54, 0.60]
	spin	0.14 [0.12, 0.16]	0.12 [0.11, 0.15]	0.13 [0.11, 0.15]	0.15 [0.13, 0.17]
Dorsal Attention	NEST	0.05 [0.04, 0.07]	0.06 [0.05, 0.08]	0.04 [0.03, 0.06]	0.06 [0.05, 0.08]
	FastGSEA	0.36 [0.33, 0.39]	0.37 [0.34, 0.40]	0.37 [0.34, 0.40]	0.36 [0.33, 0.39]
	spin	0.04 [0.03, 0.05]	0.04 [0.03, 0.06]	0.05 [0.04, 0.06]	0.04 [0.03, 0.05]
Ventral Attention	NEST	0.06 [0.04, 0.07]	0.04 [0.03, 0.06]	0.04 [0.03, 0.06]	0.06 [0.05, 0.08]
	FastGSEA	0.37 [0.34, 0.40]	0.38 [0.35, 0.41]	0.39 [0.36, 0.42]	0.39 [0.36, 0.42]
	spin	0.05 [0.04, 0.06]	0.06 [0.05, 0.07]	0.04 [0.03, 0.06]	0.05 [0.04, 0.07]
Limbic	NEST	0.05 [0.04, 0.07]	0.04 [0.03, 0.06]	0.06 [0.05, 0.08]	0.05 [0.04, 0.07]
	FastGSEA	0.45 [0.42, 0.48]	0.50 [0.47, 0.53]	0.49 [0.46, 0.52]	0.52 [0.49, 0.55]
	spin	0.09 [0.07, 0.11]	0.09 [0.07, 0.11]	0.11 [0.09, 0.13]	0.09 [0.07, 0.11]
Frontoparietal	NEST	0.05 [0.04, 0.06]	0.05 [0.04, 0.06]	0.05 [0.04, 0.07]	0.06 [0.04, 0.07]
	FastGSEA	0.37 [0.34, 0.40]	0.34 [0.31, 0.37]	0.38 [0.35, 0.41]	0.35 [0.32, 0.38]
	spin	0.07 [0.06, 0.09]	0.07 [0.06, 0.09]	0.07 [0.06, 0.09]	0.08 [0.06, 0.10]
Default	NEST	0.05 [0.04, 0.06]	0.05 [0.04, 0.06]	0.05 [0.04, 0.07]	0.05 [0.04, 0.06]
	FastGSEA	0.39 [0.36, 0.42]	0.41 [0.38, 0.44]	0.43 [0.40, 0.46]	0.43 [0.40, 0.46]
	spin	0.09 [0.08, 0.11]	0.10 [0.08, 0.12]	0.09 [0.08, 0.11]	0.10 [0.08, 0.11]

Table C.1(c) Type I error rates for cortical thickness-age × sex associations (simulated under H_0). Note: these results correspond to the top right panel of Figure **??**.

		$N_{sub} = 50$	$N_{sub} = 100$	$N_{sub} = 200$	$N_{sub} = 300$
Visual	NEST	0.05 [0.04, 0.07]	0.04 [0.03, 0.06]	0.05 [0.03, 0.06]	0.05 [0.04, 0.07]
	FastGSEA	0.44 [0.41, 0.47]	0.47 [0.44, 0.50]	0.50 [0.47, 0.53]	0.48 [0.45, 0.51]
	spin	0.07 [0.06, 0.09]	0.08 [0.06, 0.10]	0.07 [0.06, 0.09]	0.07 [0.05, 0.08]
Somatomotor	NEST	0.05 [0.04, 0.07]	0.04 [0.03, 0.05]	0.05 [0.04, 0.06]	0.04 [0.03, 0.06]
	FastGSEA	0.51 [0.48, 0.54]	0.53 [0.50, 0.56]	0.51 [0.48, 0.54]	0.52 [0.49, 0.55]
	spin	0.14 [0.12, 0.17]	0.15 [0.13, 0.17]	0.12 [0.10, 0.14]	0.13 [0.11, 0.16]
Dorsal Attention	NEST	0.05 [0.04, 0.06]	0.05 [0.04, 0.07]	0.05 [0.04, 0.07]	0.04 [0.03, 0.05]
	FastGSEA	0.35 [0.32, 0.38]	0.38 [0.35, 0.41]	0.39 [0.36, 0.42]	0.36 [0.33, 0.39]
	spin	0.05 [0.04, 0.06]	0.05 [0.04, 0.07]	0.06 [0.05, 0.08]	0.05 [0.04, 0.07]
Ventral Attention	NEST	0.04 [0.03, 0.05]	0.05 [0.04, 0.07]	0.05 [0.04, 0.07]	0.05 [0.04, 0.06]
	FastGSEA	0.30 [0.28, 0.33]	0.29 [0.26, 0.32]	0.33 [0.30, 0.36]	0.32 [0.29, 0.35]
	spin	0.04 [0.03, 0.06]	0.04 [0.03, 0.06]	0.05 [0.04, 0.06]	0.05 [0.04, 0.07]
Limbic	NEST	0.05 [0.04, 0.07]	0.04 [0.03, 0.06]	0.04 [0.03, 0.06]	0.04 [0.03, 0.05]
	FastGSEA	0.46 [0.43, 0.49]	0.48 [0.45, 0.51]	0.49 [0.46, 0.52]	0.46 [0.43, 0.49]
	spin	0.09 [0.07, 0.11]	0.11 [0.09, 0.13]	0.11 [0.09, 0.13]	0.10 [0.08, 0.12]
Frontoparietal	NEST	0.05 [0.04, 0.06]	0.04 [0.03, 0.06]	0.05 [0.04, 0.06]	0.04 [0.03, 0.05]
	FastGSEA	0.34 [0.31, 0.37]	0.30 [0.27, 0.33]	0.35 [0.32, 0.38]	0.34 [0.31, 0.37]
	spin	0.07 [0.06, 0.09]	0.06 [0.04, 0.07]	0.06 [0.04, 0.07]	0.09 [0.07, 0.11]
Default	NEST	0.05 [0.04, 0.06]	0.04 [0.03, 0.05]	0.04 [0.03, 0.06]	0.06 [0.05, 0.08]
	FastGSEA	0.37 [0.34, 0.40]	0.39 [0.36, 0.42]	0.38 [0.35, 0.41]	0.39 [0.36, 0.42]
	spin	0.08 [0.06, 0.10]	0.10 [0.09, 0.13]	0.09 [0.08, 0.11]	0.09 [0.08, 0.11]

Table C.1(d) Type I error rates for n-back-age associations (simulated under H_0).
Note: these results correspond to the bottom left panel of Figure **??**.

		$N_{sub} = 50$	$N_{sub} = 100$	$N_{sub} = 200$	$N_{sub} = 300$
Visual	NEST	0.04 [0.03, 0.06]	0.04 [0.03, 0.05]	0.04 [0.03, 0.05]	0.05 [0.04, 0.07]
	FastGSEA	0.72 [0.70, 0.75]	0.78 [0.75, 0.80]	0.83 [0.80, 0.85]	0.78 [0.76, 0.81]
	spin	0.14 [0.12, 0.16]	0.13 [0.11, 0.15]	0.14 [0.12, 0.16]	0.14 [0.12, 0.17]
Somatomotor	NEST	0.04 [0.03, 0.05]	0.05 [0.04, 0.06]	0.05 [0.04, 0.07]	0.05 [0.04, 0.06]
	FastGSEA	0.76 [0.73, 0.78]	0.80 [0.78, 0.83]	0.82 [0.79, 0.84]	0.81 [0.79, 0.83]
	spin	0.35 [0.32, 0.38]	0.35 [0.32, 0.38]	0.34 [0.31, 0.37]	0.32 [0.29, 0.35]
Dorsal Attention	NEST	0.04 [0.03, 0.05]	0.04 [0.03, 0.06]	0.05 [0.04, 0.07]	0.05 [0.04, 0.06]
	FastGSEA	0.71 [0.68, 0.74]	0.75 [0.72, 0.77]	0.75 [0.72, 0.77]	0.77 [0.75, 0.80]
	spin	0.30 [0.28, 0.33]	0.30 [0.27, 0.33]	0.32 [0.29, 0.35]	0.32 [0.29, 0.35]
Ventral Attention	NEST	0.05 [0.04, 0.07]	0.06 [0.05, 0.08]	0.04 [0.03, 0.05]	0.06 [0.05, 0.08]
	FastGSEA	0.65 [0.62, 0.68]	0.73 [0.70, 0.76]	0.72 [0.69, 0.74]	0.75 [0.72, 0.78]
	spin	0.24 [0.22, 0.27]	0.28 [0.25, 0.31]	0.26 [0.23, 0.28]	0.26 [0.23, 0.28]
Limbic	NEST	0.06 [0.05, 0.08]	0.05 [0.04, 0.06]	0.04 [0.03, 0.06]	0.04 [0.03, 0.06]
	FastGSEA	0.69 [0.66, 0.72]	0.75 [0.72, 0.77]	0.79 [0.77, 0.82]	0.78 [0.76, 0.81]
	spin	0.18 [0.16, 0.21]	0.19 [0.17, 0.22]	0.23 [0.20, 0.26]	0.21 [0.18, 0.23]
Frontoparietal	NEST	0.05 [0.04, 0.06]	0.04 [0.03, 0.05]	0.06 [0.05, 0.08]	0.05 [0.04, 0.06]
	FastGSEA	0.73 [0.71, 0.76]	0.77 [0.74, 0.79]	0.79 [0.76, 0.81]	0.80 [0.77, 0.82]
	spin	0.43 [0.40, 0.46]	0.42 [0.39, 0.45]	0.41 [0.38, 0.44]	0.39 [0.36, 0.42]
Default	NEST	0.06 [0.04, 0.07]	0.06 [0.05, 0.08]	0.06 [0.05, 0.08]	0.05 [0.04, 0.06]
	FastGSEA	0.69 [0.66, 0.72]	0.74 [0.72, 0.77]	0.71 [0.68, 0.74]	0.73 [0.70, 0.76]
	spin	0.35 [0.32, 0.38]	0.35 [0.32, 0.38]	0.34 [0.31, 0.37]	0.33 [0.31, 0.36]

Table C.1(e) Type I error rates for n-back-sex associations (simulated under H_0).
Note: these results correspond to the bottom middle panel of Figure **??**.

		$N_{sub} = 50$	$N_{sub} = 100$	$N_{sub} = 200$	$N_{sub} = 300$
Visual	NEST	0.04 [0.03, 0.05]	0.05 [0.04, 0.07]	0.05 [0.04, 0.07]	0.05 [0.04, 0.07]
	FastGSEA	0.70 [0.67, 0.73]	0.72 [0.69, 0.75]	0.76 [0.74, 0.79]	0.75 [0.72, 0.78]
	spin	0.10 [0.09, 0.13]	0.13 [0.11, 0.15]	0.15 [0.13, 0.18]	0.14 [0.12, 0.16]
Somatomotor	NEST	0.05 [0.03, 0.06]	0.05 [0.04, 0.06]	0.06 [0.04, 0.07]	0.05 [0.04, 0.07]
	FastGSEA	0.74 [0.71, 0.77]	0.75 [0.72, 0.78]	0.74 [0.72, 0.77]	0.76 [0.74, 0.79]
	spin	0.32 [0.29, 0.35]	0.34 [0.32, 0.37]	0.33 [0.30, 0.36]	0.34 [0.31, 0.37]
Dorsal Attention	NEST	0.04 [0.03, 0.05]	0.05 [0.04, 0.06]	0.05 [0.04, 0.06]	0.04 [0.03, 0.06]
	FastGSEA	0.67 [0.64, 0.70]	0.69 [0.66, 0.72]	0.70 [0.68, 0.73]	0.72 [0.69, 0.74]
	spin	0.30 [0.27, 0.33]	0.30 [0.27, 0.33]	0.32 [0.29, 0.35]	0.29 [0.27, 0.32]
Ventral Attention	NEST	0.04 [0.03, 0.06]	0.05 [0.04, 0.06]	0.05 [0.03, 0.06]	0.06 [0.05, 0.08]
	FastGSEA	0.62 [0.59, 0.65]	0.69 [0.66, 0.71]	0.67 [0.64, 0.70]	0.69 [0.66, 0.72]
	spin	0.24 [0.21, 0.27]	0.25 [0.22, 0.27]	0.22 [0.20, 0.25]	0.24 [0.21, 0.26]
Limbic	NEST	0.05 [0.04, 0.07]	0.06 [0.04, 0.07]	0.05 [0.04, 0.07]	0.04 [0.03, 0.06]
	FastGSEA	0.76 [0.73, 0.78]	0.74 [0.71, 0.77]	0.77 [0.74, 0.79]	0.75 [0.72, 0.77]
	spin	0.22 [0.19, 0.24]	0.21 [0.19, 0.24]	0.19 [0.17, 0.22]	0.20 [0.18, 0.23]
Frontoparietal	NEST	0.05 [0.04, 0.06]	0.05 [0.04, 0.07]	0.06 [0.05, 0.08]	0.06 [0.05, 0.07]
	FastGSEA	0.68 [0.65, 0.70]	0.70 [0.67, 0.73]	0.71 [0.68, 0.74]	0.70 [0.67, 0.73]
	spin	0.41 [0.38, 0.44]	0.40 [0.37, 0.43]	0.43 [0.40, 0.46]	0.41 [0.38, 0.44]
Default	NEST	0.04 [0.03, 0.06]	0.05 [0.03, 0.06]	0.05 [0.04, 0.07]	0.05 [0.03, 0.06]
	FastGSEA	0.65 [0.62, 0.68]	0.70 [0.67, 0.73]	0.68 [0.65, 0.71]	0.66 [0.63, 0.69]
	spin	0.34 [0.32, 0.37]	0.35 [0.32, 0.38]	0.36 [0.33, 0.39]	0.35 [0.32, 0.38]

Table C.1(f) Type I error rates for n-back-age × sex associations (simulated under H_0).
Note: these results correspond to the bottom right panel of Figure **??**.

		$N_{sub} = 50$	$N_{sub} = 100$	$N_{sub} = 200$	$N_{sub} = 300$
Visual	NEST	0.05 [0.04, 0.07]	0.05 [0.04, 0.07]	0.05 [0.04, 0.07]	0.05 [0.04, 0.07]
	FastGSEA	0.69 [0.66, 0.72]	0.75 [0.72, 0.77]	0.75 [0.72, 0.78]	0.74 [0.71, 0.77]
	spin	0.14 [0.12, 0.16]	0.16 [0.14, 0.19]	0.15 [0.13, 0.17]	0.13 [0.11, 0.15]
Somatomotor	NEST	0.04 [0.03, 0.06]	0.05 [0.04, 0.07]	0.05 [0.04, 0.06]	0.04 [0.03, 0.06]
	FastGSEA	0.74 [0.71, 0.76]	0.76 [0.73, 0.78]	0.76 [0.73, 0.79]	0.75 [0.72, 0.77]
	spin	0.34 [0.31, 0.37]	0.32 [0.29, 0.35]	0.33 [0.30, 0.36]	0.31 [0.28, 0.34]
Dorsal Attention	NEST	0.04 [0.03, 0.06]	0.04 [0.03, 0.06]	0.06 [0.05, 0.08]	0.06 [0.04, 0.07]
	FastGSEA	0.71 [0.68, 0.74]	0.69 [0.66, 0.72]	0.72 [0.69, 0.75]	0.72 [0.69, 0.74]
	spin	0.28 [0.26, 0.31]	0.30 [0.28, 0.33]	0.29 [0.26, 0.32]	0.32 [0.29, 0.35]
Ventral Attention	NEST	0.06 [0.05, 0.07]	0.05 [0.04, 0.06]	0.05 [0.04, 0.06]	0.05 [0.03, 0.06]
	FastGSEA	0.68 [0.65, 0.71]	0.69 [0.66, 0.71]	0.67 [0.64, 0.70]	0.68 [0.65, 0.71]
	spin	0.24 [0.22, 0.27]	0.24 [0.21, 0.26]	0.25 [0.22, 0.27]	0.24 [0.21, 0.27]
Limbic	NEST	0.05 [0.04, 0.06]	0.04 [0.03, 0.06]	0.05 [0.04, 0.06]	0.04 [0.03, 0.06]
	FastGSEA	0.68 [0.65, 0.70]	0.69 [0.66, 0.72]	0.72 [0.70, 0.75]	0.72 [0.70, 0.75]
	spin	0.19 [0.17, 0.22]	0.16 [0.14, 0.19]	0.19 [0.17, 0.22]	0.20 [0.17, 0.22]
Frontoparietal	NEST	0.05 [0.03, 0.06]	0.05 [0.04, 0.06]	0.05 [0.04, 0.07]	0.05 [0.04, 0.07]
	FastGSEA	0.69 [0.66, 0.71]	0.70 [0.67, 0.72]	0.74 [0.71, 0.77]	0.71 [0.68, 0.74]
	spin	0.39 [0.36, 0.42]	0.40 [0.37, 0.43]	0.38 [0.35, 0.41]	0.38 [0.35, 0.41]
Default	NEST	0.04 [0.03, 0.06]	0.06 [0.04, 0.07]	0.04 [0.03, 0.06]	0.04 [0.03, 0.06]
	FastGSEA	0.69 [0.66, 0.71]	0.69 [0.66, 0.72]	0.70 [0.67, 0.73]	0.71 [0.68, 0.73]
	spin	0.36 [0.33, 0.39]	0.34 [0.31, 0.37]	0.33 [0.30, 0.36]	0.38 [0.35, 0.41]

Table C.2. Power simulations: enrichment of brain-phenotype associations in Yeo et al. (2011)'s seven functional networks. Power estimates and 95% confidence intervals are each based on 1000 random subsamples (size N_{sub} each) from the Philadelphia Neurodevelopmental Cohort dataset, with $K = 999$ permutations used to test enrichment of each network on each subsample. We also presented these results in the form of Figure **??**.

Table C.2(a) Power of NEST in simulation studies testing enrichment of age effects on cortical thickness and n-back in the seven functional networks.

	$N_{sub} = 50$	$N_{sub} = 100$	$N_{sub} = 200$	$N_{sub} = 300$
Cortical thickness				
Visual	0.068	0.050	0.027	0.015
	[0.054-0.085]	[0.038-0.066]	[0.019-0.039]	[0.009-0.025]
Somatomotor	0.058	0.046	0.021	0.010
	[0.045-0.074]	[0.035-0.061]	[0.014-0.032]	[0.005-0.019]
Dorsal Attention	0.703	0.912	0.995	1.000
	[0.674-0.730]	[0.893-0.928]	[0.988-0.998]	[0.995-1.000]
Ventral Attention	0.367	0.492	0.744	0.915
	[0.338-0.397]	[0.461-0.523]	[0.716-0.770]	[0.896-0.931]
Limbic	0.145	0.224	0.204	0.139
	[0.125-0.168]	[0.199-0.251]	[0.180-0.230]	[0.119-0.162]
Frontoparietal	0.590	0.764	0.936	0.988
	[0.559-0.620]	[0.737-0.789]	[0.919-0.950]	[0.979-0.993]
Default	0.834	0.983	1.000	1.000
	[0.810-0.856]	[0.973-0.990]	[0.995-1.000]	[0.995-1.000]
N-back				
Visual	0.035	0.023	0.006	0.002
	[0.025-0.049]	[0.015-0.035]	[0.002-0.013]	[0.000-0.008]
Somatomotor	0.063	0.056	0.074	0.067
	[0.050-0.080]	[0.043-0.072]	[0.059-0.092]	[0.053-0.084]
Dorsal Attention	0.082	0.131	0.217	0.294
	[0.067-0.101]	[0.112-0.153]	[0.193-0.244]	[0.267-0.323]
Ventral Attention	0.043	0.026	0.020	0.013
	[0.032-0.058]	[0.018-0.038]	[0.013-0.031]	[0.007-0.022]
Limbic	0.078	0.070	0.053	0.056
	[0.063-0.096]	[0.056-0.088]	[0.041-0.069]	[0.043-0.072]
Frontoparietal	0.100	0.137	0.253	0.368
	[0.083-0.120]	[0.117-0.160]	[0.227-0.281]	[0.339-0.398]
Default	0.028	0.027	0.015	0.004
	[0.019-0.040]	[0.019-0.039]	[0.009-0.025]	[0.001-0.011]

Table C.2(b) Power of NEST in simulation studies testing enrichment of sex effects on cortical thickness and n-back in the seven functional networks.

	$N_{sub} = 50$	$N_{sub} = 100$	$N_{sub} = 200$	$N_{sub} = 300$
Cortical thickness				
Visual	0.128	0.211	0.419	0.573
	[0.109-0.150]	[0.187-0.237]	[0.389-0.450]	[0.542-0.603]
Somatomotor	0.031	0.047	0.034	0.025
	[0.022-0.044]	[0.035-0.062]	[0.024-0.047]	[0.017-0.037]
Dorsal Attention	0.038	0.027	0.022	0.014
	[0.028-0.052]	[0.019-0.039]	[0.014-0.033]	[0.008-0.024]
Ventral Attention	0.128	0.193	0.311	0.463
	[0.109-0.150]	[0.170-0.219]	[0.283-0.340]	[0.432-0.494]
Limbic	0.027	0.040	0.022	0.010
	[0.019-0.039]	[0.029-0.054]	[0.014-0.033]	[0.005-0.019]
Frontoparietal	0.137	0.263	0.532	0.719
	[0.117-0.160]	[0.237-0.291]	[0.501-0.563]	[0.690-0.746]
Default	0.074	0.116	0.185	0.246
	[0.059-0.092]	[0.098-0.137]	[0.162-0.210]	[0.220-0.274]
N-back				
Visual	0.034	0.039	0.025	0.011
	[0.024-0.047]	[0.029-0.053]	[0.017-0.037]	[0.006-0.020]
Somatomotor	0.059	0.066	0.103	0.108
	[0.046-0.076]	[0.052-0.083]	[0.086-0.124]	[0.090-0.129]
Dorsal Attention	0.049	0.047	0.021	0.013
	[0.037-0.064]	[0.035-0.062]	[0.014-0.032]	[0.007-0.022]
Ventral Attention	0.045	0.049	0.068	0.080
	[0.034-0.060]	[0.037-0.064]	[0.054-0.085]	[0.065-0.099]
Limbic	0.042	0.037	0.032	0.025
	[0.031-0.056]	[0.027-0.051]	[0.023-0.045]	[0.017-0.037]
Frontoparietal	0.085	0.094	0.151	0.215
	[0.069-0.104]	[0.077-0.114]	[0.130-0.175]	[0.191-0.242]
Default	0.053	0.068	0.084	0.076
	[0.041-0.069]	[0.054-0.085]	[0.068-0.103]	[0.061-0.094]

Table C.2(c) Power of NEST in simulation studies testing enrichment of age × sex effects on cortical thickness and n-back in the seven functional networks.

	$N_{sub} = 50$	$N_{sub} = 100$	$N_{sub} = 200$	$N_{sub} = 300$
Cortical thickness				
Visual	0.102<br>[0.085-0.122]	0.102<br>[0.085-0.122]	0.093<br>[0.077-0.113]	0.055<br>[0.042-0.071]
Somatomotor	0.111<br>[0.093-0.132]	0.119<br>[0.100-0.141]	0.150<br>[0.129-0.174]	0.166<br>[0.144-0.190]
Dorsal Attention	0.139<br>[0.119-0.162]	0.168<br>[0.146-0.193]	0.301<br>[0.273-0.330]	0.409<br>[0.379-0.440]
Ventral Attention	0.220<br>[0.195-0.247]	0.319<br>[0.291-0.349]	0.566<br>[0.535-0.596]	0.738<br>[0.710-0.764]
Limbic	0.073<br>[0.058-0.091]	0.069<br>[0.055-0.087]	0.061<br>[0.048-0.078]	0.041<br>[0.030-0.055]
Frontoparietal	0.112<br>[0.094-0.133]	0.136<br>[0.116-0.159]	0.177<br>[0.155-0.202]	0.204<br>[0.180-0.230]
Default	0.134<br>[0.114-0.157]	0.172<br>[0.150-0.197]	0.285<br>[0.258-0.314]	0.370<br>[0.341-0.400]
N-back				
Visual	0.076<br>[0.061-0.094]	0.066<br>[0.052-0.083]	0.057<br>[0.044-0.073]	0.056<br>[0.043-0.072]
Somatomotor	0.085<br>[0.069-0.104]	0.073<br>[0.058-0.091]	0.071<br>[0.057-0.089]	0.051<br>[0.039-0.067]
Dorsal Attention	0.058<br>[0.045-0.074]	0.064<br>[0.050-0.081]	0.038<br>[0.028-0.052]	0.022<br>[0.014-0.033]
Ventral Attention	0.093<br>[0.077-0.113]	0.083<br>[0.067-0.102]	0.086<br>[0.070-0.105]	0.096<br>[0.079-0.116]
Limbic	0.121<br>[0.102-0.143]	0.115<br>[0.097-0.136]	0.132<br>[0.112-0.155]	0.164<br>[0.142-0.188]
Frontoparietal	0.105<br>[0.087-0.126]	0.125<br>[0.106-0.147]	0.137<br>[0.117-0.160]	0.131<br>[0.112-0.153]
Default	0.069<br>[0.055-0.087]	0.052<br>[0.040-0.068]	0.034<br>[0.024-0.047]	0.026<br>[0.018-0.038]

Table C.3. Power [95% CI] of NEST in enrichment tests of brain-phenotype associations when using data at different resolutions. In our original power simulations (see Figure **??** and Table C.2), we used vertex-level fsaverage5 measurements, totalling $V = 18,715$ vertices across both hemispheres, after the medial wall. Here, we consider different resolutions of the data based on Schaefer et al. (2018)'s parcellations with $V = 100, 200, 500,$ and 1000 parcels across both hemispheres.

Table C.3(a) Cortical thickness-age effects: power at different resolutions by sub-sample size.

	Visual	Somatomotor	Dorsal Attention	Ventral Attention	Limbic	Frontoparietal	Default
$N_{sub} = 50$							
$V = 100$	0.011	0.018	0.080	0.146	0.057	0.232	0.192
	[0.006-0.020]	[0.011-0.029]	[0.065-0.099]	[0.125-0.169]	[0.044-0.073]	[0.207-0.259]	[0.169-0.218]
$V = 200$	0.012	0.008	0.160	0.187	0.036	0.240	0.286
	[0.007-0.021]	[0.004-0.016]	[0.139-0.184]	[0.164-0.212]	[0.026-0.050]	[0.215-0.267]	[0.259-0.315]
$V = 500$	0.019	0.007	0.263	0.327	0.025	0.280	0.517
	[0.012-0.030]	[0.003-0.015]	[0.237-0.291]	[0.299-0.357]	[0.017-0.037]	[0.253-0.309]	[0.486-0.548]
$V = 1000$	0.024	0.020	0.301	0.296	0.030	0.369	0.620
	[0.016-0.036]	[0.013-0.031]	[0.273-0.330]	[0.269-0.325]	[0.021-0.043]	[0.340-0.399]	[0.589-0.650]
$N_{sub} = 100$							
$V = 100$	0.008	0.032	0.139	0.163	0.101	0.417	0.243
	[0.004-0.016]	[0.023-0.045]	[0.119-0.162]	[0.141-0.187]	[0.084-0.121]	[0.387-0.448]	[0.217-0.271]
$V = 200$	0.005	0.003	0.253	0.275	0.063	0.449	0.522
	[0.002-0.012]	[0.001-0.009]	[0.227-0.281]	[0.248-0.304]	[0.050-0.080]	[0.418-0.480]	[0.491-0.553]
$V = 500$	0.007	0.003	0.429	0.623	0.026	0.514	0.818
	[0.003-0.015]	[0.001-0.009]	[0.399-0.460]	[0.593-0.652]	[0.018-0.038]	[0.483-0.545]	[0.793-0.841]
$V = 1000$	0.004	0.005	0.487	0.449	0.019	0.546	0.921
	[0.001-0.011]	[0.002-0.012]	[0.456-0.518]	[0.418-0.480]	[0.012-0.030]	[0.515-0.577]	[0.902-0.936]
$N_{sub} = 200$							
$V = 100$	0.003	0.014	0.185	0.172	0.153	0.640	0.364
	[0.001-0.009]	[0.008-0.024]	[0.162-0.210]	[0.150-0.197]	[0.132-0.177]	[0.610-0.669]	[0.335-0.394]
$V = 200$	0.001	0.000	0.463	0.499	0.085	0.873	0.816
	[0.000-0.006]	[0.000-0.005]	[0.432-0.494]	[0.468-0.530]	[0.069-0.104]	[0.851-0.892]	[0.791-0.839]
$V = 500$	0.000	0.000	0.748	0.962	0.053	0.915	0.997
	[0.000-0.005]	[0.000-0.005]	[0.720-0.774]	[0.948-0.972]	[0.041-0.069]	[0.896-0.931]	[0.991-0.999]
$V = 1000$	0.001	0.000	0.769	0.813	0.013	0.892	1.000
	[0.000-0.006]	[0.000-0.005]	[0.742-0.794]	[0.788-0.836]	[0.007-0.022]	[0.871-0.910]	[0.995-1.000]
$N_{sub} = 300$							
$V = 100$	0.000	0.003	0.226	0.209	0.132	0.776	0.427
	[0.000-0.005]	[0.001-0.009]	[0.201-0.253]	[0.185-0.235]	[0.112-0.155]	[0.749-0.801]	[0.397-0.458]
$V = 200$	0.000	0.000	0.612	0.751	0.065	0.988	0.956
	[0.000-0.005]	[0.000-0.005]	[0.581-0.642]	[0.723-0.777]	[0.051-0.082]	[0.979-0.993]	[0.941-0.967]
$V = 500$	0.000	0.000	0.941	0.999	0.054	0.997	1.000
	[0.000-0.005]	[0.000-0.005]	[0.924-0.954]	[0.994-1.000]	[0.042-0.070]	[0.991-0.999]	[0.995-1.000]
$V = 1000$	0.000	0.000	0.943	0.973	0.010	0.991	1.000
	[0.000-0.005]	[0.000-0.005]	[0.927-0.956]	[0.961-0.981]	[0.005-0.019]	[0.983-0.995]	[0.995-1.000]

Table C.3(b) N-back-age effects: power at different resolutions by sub-sample size.

	Visual	Somatomotor	Dorsal Attention	Ventral Attention	Limbic	Frontoparietal	Default
$N_{sub} = 50$							
$V = 100$	0.033	0.077	0.075	0.043	0.063	0.056	0.026
	[0.024-0.046]	[0.062-0.095]	[0.060-0.093]	[0.032-0.058]	[0.050-0.080]	[0.043-0.072]	[0.018-0.038]
$V = 200$	0.030	0.055	0.076	0.050	0.065	0.081	0.022
	[0.021-0.043]	[0.042-0.071]	[0.061-0.094]	[0.038-0.066]	[0.051-0.082]	[0.066-0.100]	[0.014-0.033]
$V = 500$	0.028	0.056	0.084	0.043	0.071	0.088	0.021
	[0.019-0.040]	[0.043-0.072]	[0.068-0.103]	[0.032-0.058]	[0.057-0.089]	[0.072-0.107]	[0.014-0.032]
$V = 1000$	0.030	0.061	0.063	0.044	0.067	0.098	0.026
	[0.021-0.043]	[0.048-0.078]	[0.050-0.080]	[0.033-0.059]	[0.053-0.084]	[0.081-0.118]	[0.018-0.038]
$N_{sub} = 100$							
$V = 100$	0.019	0.079	0.093	0.047	0.056	0.055	0.019
	[0.012-0.030]	[0.064-0.098]	[0.077-0.113]	[0.035-0.062]	[0.043-0.072]	[0.042-0.071]	[0.012-0.030]
$V = 200$	0.023	0.054	0.096	0.032	0.058	0.103	0.023
	[0.015-0.035]	[0.042-0.070]	[0.079-0.116]	[0.023-0.045]	[0.045-0.074]	[0.086-0.124]	[0.015-0.035]
$V = 500$	0.022	0.057	0.133	0.024	0.066	0.122	0.027
	[0.014-0.033]	[0.044-0.073]	[0.113-0.156]	[0.016-0.036]	[0.052-0.083]	[0.103-0.144]	[0.019-0.039]
$V = 1000$	0.017	0.055	0.102	0.036	0.067	0.129	0.025
	[0.010-0.027]	[0.042-0.071]	[0.085-0.122]	[0.026-0.050]	[0.053-0.084]	[0.110-0.151]	[0.017-0.037]
$N_{sub} = 200$							
$V = 100$	0.008	0.095	0.129	0.029	0.039	0.069	0.010
	[0.004-0.016]	[0.078-0.115]	[0.110-0.151]	[0.020-0.042]	[0.029-0.053]	[0.055-0.087]	[0.005-0.019]
$V = 200$	0.008	0.062	0.146	0.028	0.032	0.139	0.006
	[0.004-0.016]	[0.049-0.079]	[0.125-0.169]	[0.019-0.040]	[0.023-0.045]	[0.119-0.162]	[0.002-0.013]
$V = 500$	0.006	0.064	0.221	0.018	0.046	0.210	0.013
	[0.002-0.013]	[0.050-0.081]	[0.196-0.248]	[0.011-0.029]	[0.035-0.061]	[0.186-0.236]	[0.007-0.022]
$V = 1000$	0.005	0.075	0.145	0.021	0.048	0.244	0.015
	[0.002-0.012]	[0.060-0.093]	[0.125-0.168]	[0.014-0.032]	[0.036-0.063]	[0.218-0.272]	[0.009-0.025]
$N_{sub} = 300$							
$V = 100$	0.005	0.105	0.177	0.026	0.025	0.092	0.007
	[0.002-0.012]	[0.087-0.126]	[0.155-0.202]	[0.018-0.038]	[0.017-0.037]	[0.076-0.112]	[0.003-0.015]
$V = 200$	0.001	0.060	0.168	0.013	0.035	0.202	0.005
	[0.000-0.006]	[0.047-0.077]	[0.146-0.193]	[0.007-0.022]	[0.025-0.049]	[0.178-0.228]	[0.002-0.012]
$V = 500$	0.002	0.061	0.298	0.014	0.040	0.306	0.010
	[0.000-0.008]	[0.048-0.078]	[0.270-0.327]	[0.008-0.024]	[0.029-0.054]	[0.278-0.335]	[0.005-0.019]
$V = 1000$	0.000	0.081	0.188	0.006	0.045	0.371	0.008
	[0.000-0.005]	[0.066-0.100]	[0.165-0.213]	[0.002-0.013]	[0.034-0.060]	[0.342-0.401]	[0.004-0.016]

Table C.4. In a post-hoc analysis, we apply NEST to assess enrichment of brain-phenotype associations in alternative network partitions, using data from the Philadelphia Neurodevelopmental Cohort (Satterthwaite et al. 2014). Unadjusted p-values from tests of enrichment within the full PNC sample (after exclusion criteria) are reported below. (Given that these analyses were conducted post-hoc, we do not adjust for multiple comparisons.) FreeSurfer annotation label names are included for both versions of network partitions (with the first number indicating whether the network is from the 7- or 17-network version and the number after the underscore indicating the label within the 7- or 17-network partition).

	cortical thickness			n-back		
	age	sex	age × sex	age	sex	age × sex
**Visual**						
`7Networks_1`*	0.406	<0.001	0.719	0.769	0.796	0.500
`17Networks_1`	0.479	0.052	0.549	0.546	0.554	0.315
`17Networks_2`	0.783	0.002	0.562	0.871	0.925	0.695
**Somatomotor**						
`7Networks_2`*	0.306	0.453	0.242	0.165	0.132	0.689
`17Networks_3`	0.182	0.131	0.829	0.677	0.934	0.538
`17Networks_4`	0.915	0.268	0.057	0.036	<0.001	0.599
**Dorsal Attention**						
`7Networks_3`*	<0.001	0.976	0.018	0.024	0.717	0.985
`17Networks_5`	<0.001	0.255	0.174	0.366	0.900	0.832
`17Networks_6`	0.003	0.280	0.019	0.045	0.959	0.772
**Ventral Attention**						
`7Networks_4`*	<0.001	0.007	0.001	0.439	0.081	0.313
`17Networks_7`	<0.001	<0.001	0.006	0.365	0.004	0.518
`17Networks_8`	0.029	0.126	0.207	0.001	0.004	0.084
**Limbic**						
`7Networks_5`*	0.255	0.600	0.735	0.354	0.581	0.328
`17Networks_9`	<0.001	0.368	0.382	0.840	0.296	0.800
`17Networks_10`	0.134	0.082	0.406	0.092	0.940	0.048
**Frontoparietal**						
`7Networks_6`*	<0.001	<0.001	0.286	0.003	0.020	0.225
`17Networks_11`	<0.001	0.359	0.123	0.795	<0.001	0.233
`17Networks_12`	<0.001	0.374	0.078	0.003	0.468	0.549
`17Networks_13`	0.011	<0.001	0.998	0.009	0.061	0.320
`17Networks_14`	<0.001	0.250	0.034	0.256	0.346	0.435
**Default**						
`7Networks_7`*	<0.001	0.014	0.072	0.990	0.117	0.815
`17Networks_15`	0.036	0.181	0.053	0.310	0.679	0.818
`17Networks_16`	<0.001	0.004	0.083	0.590	0.021	0.782
`17Networks_17`	<0.001	0.112	0.108	0.504	0.787	0.763

*Results for networks from Yeo et al. (2011)'s 7-network partition were also presented in Figure ??.

Appendix D Computational efficiency

Table D.5. Average (SD) computation times in seconds over repeated tests of enrichment in different sample sizes, at different spatial resolutions, and using different methods for quantifying $T(v)$ (statistic of brain-phenotype associations). Simulations are conducted using our implementation of NEST in R (see https://smweinst.github.io/nest-method/), using three cores per simulation (on 24-core/48-thread Intel Xeon Gold processors) for parallelization across permutations. We use Schaefer et al. (2018)'s parcellations to down-sample participant-level fsaverage5 maps (vertex-level maps with $V = 18,715$ across both hemispheres, after removing the medial wall) to various lower resolutions ($V = 100$, 200, 500, $1,000$). Summary statistics are taken over 2,000 simulations involving age effects on cortical thickness and n-back (1,000 simulations per modality). In each simulation, the recorded time includes time to test enrichment in all seven networks from Yeo et al. However, the most time-intensive portion of these analyses— estimation of $T(v)$ at all vertices in both the observed and all permuted datasets— occurs only once per (observed or permuted) sample, given that the same map of $T(v)$'s is used to compute enrichment scores for different networks. Rows labeled "GAM" estimate $T(v)$ using the statistic described in Appendix B. Rows labeled "LM" involves fitting a multiple linear regression model with each brain measurement as the outcome regressed on age, sex, and their interaction, and using estimated age effects at each vertex as $T(v)$.

	Parcellated brain maps (Schaefer et al. 2018)				Vertex-level
	$V = 100$	$V = 200$	$V = 500$	$V = 1,000$	fsaverage5
$N_{sub} = 50$					
GAM	3.38 (0.60)	4.91 (0.71)	8.88 (0.94)	15.17 (1.69)	175.60 (45.00)
LM	2.15 (0.36)	3.41 (0.49)	7.05 (0.76)	12.53 (1.40)	156.43 (40.68)
$N_{sub} = 100$					
GAM	3.50 (0.61)	5.06 (0.69)	9.10 (0.94)	15.55 (1.69)	173.70 (44.37)
LM	2.21 (0.38)	3.54 (0.49)	7.18 (0.75)	12.83 (1.39)	154.16 (40.70)
$N_{sub} = 200$					
GAM	3.82 (0.65)	5.38 (0.72)	9.47 (0.98)	16.21 (1.72)	217.52 (21.96)
LM	2.38 (0.39)	3.69 (0.51)	7.42 (0.78)	13.25 (1.40)	193.16 (22.18)
$N_{sub} = 300$					
GAM	4.04 (0.67)	5.50 (0.69)	9.66 (0.94)	16.77 (1.76)	222.84 (24.46)
LM	2.50 (0.40)	3.72 (0.49)	7.53 (0.76)	13.80 (1.46)	198.30 (23.97)

References

Alexander-Bloch, Aaron F, Haochang Shou, Siyuan Liu, Theodore D Satterthwaite, David C Glahn, Russell T Shinohara, Simon N Vandekar, and Armin Raznahan (2018). "On testing for spatial correspondence between maps of human brain structure and function". In: *Neuroimage* 178. Publisher: Elsevier, pp. 540–551.

Ciric, Rastko, Adon FG Rosen, Guray Erus, Matthew Cieslak, Azeez Adebimpe, Philip A Cook, Danielle S Bassett, Christos Davatzikos, Daniel H Wolf, and Theodore D Satterthwaite (2018). "Mitigating head motion artifact in functional connectivity MRI". In: *Nature protocols* 13.12. Publisher: Nature Publishing Group UK London, pp. 2801–2826.

Dale, Anders M, Bruce Fischl, and Martin I Sereno (1999). "Cortical surface-based analysis: I. Segmentation and surface reconstruction". In: *Neuroimage* 9.2. Publisher: Elsevier, pp. 179–194.

Korotkevich, Gennady, Vladimir Sukhov, Nikolay Budin, Boris Shpak, Maxim N Artyomov, and Alexey Sergushichev (2016). "Fast gene set enrichment analysis". In: *BioRxiv*. Publisher: Cold Spring Harbor Laboratory, p. 060012.

Park, Min Tae M, Armin Raznahan, Philip Shaw, Nitin Gogtay, Jason P Lerch, and M Mallar Chakravarty (2018). "Neuroanatomical phenotypes in mental illness: identifying convergent and divergent cortical phenotypes across autism, ADHD and schizophrenia". In: *Journal of Psychiatry and Neuroscience* 43.3. Publisher: Journal of Psychiatry and Neuroscience, pp. 201–212.

Ragland, J Daniel, Bruce I Turetsky, Ruben C Gur, Faith Gunning-Dixon, Travis Turner, Lee Schroeder, Robin Chan, and Raquel E Gur (2002). "Working memory for complex figures: an fMRI comparison of letter and fractal n-back tasks." In: *Neuropsychology* 16.3. Publisher: American Psychological Association, p. 370.

Rosen, Adon FG, David R Roalf, Kosha Ruparel, Jason Blake, Kevin Seelaus, Lakshmi P Villa, Rastko Ciric, Philip A Cook, Christos Davatzikos, Mark A Elliott, and others (2018). "Quantitative assessment of structural image quality". In: *Neuroimage* 169. Publisher: Elsevier, pp. 407–418.

Satterthwaite, Theodore D, John J Connolly, Kosha Ruparel, Monica E Calkins, Chad Jackson, Mark A Elliott, David R Roalf, Ryan Hopson, Karthik Prabhakaran, Meckenzie Behr, and others (2016). "The Philadelphia Neurodevelopmental Cohort: A publicly available resource for the study of normal and abnormal brain development in youth". In: *Neuroimage* 124. Publisher: Elsevier, pp. 1115–1119.

Satterthwaite, Theodore D, Mark A Elliott, Kosha Ruparel, James Loughead, Karthik Prabhakaran, Monica E Calkins, Ryan Hopson, Chad Jackson, Jack Keefe, Marisa Riley, and others (2014). "Neuroimaging of the Philadelphia neurodevelopmental cohort". In: *Neuroimage* 86. Publisher: Elsevier, pp. 544–553.

Schaefer, Alexander, Ru Kong, Evan M Gordon, Timothy O Laumann, Xi-Nian Zuo, Avram J Holmes, Simon B Eickhoff, and BT Thomas Yeo (2018). "Local-global parcellation of the human cerebral cortex from intrinsic functional connectivity MRI". In: *Cerebral cortex* 28.9. Publisher: Oxford University Press, pp. 3095–3114.

Weinstein, Sarah M, Simon N Vandekar, Azeez Adebimpe, Tinashe M Tapera, Timothy Robert-Fitzgerald, Ruben C Gur, Raquel E Gur, Armin Raznahan, Theodore D Satterthwaite, Aaron F Alexander-Bloch, and others (2021). "A simple permutation-based test of intermodal correspondence". In: *Human brain mapping* 42.16. Publisher: Wiley Online Library, pp. 5175–5187.

Yeo, BT Thomas, Fenna M Krienen, Jorge Sepulcre, Mert R Sabuncu, Danial Lashkari, Marisa Hollinshead, Joshua L Roffman, Jordan W Smoller, Lilla Zöllei, Jonathan R Polimeni, and others (2011). "The organization of the human cerebral cortex estimated by intrinsic functional connectivity". In: *Journal of neurophysiology*. Publisher: American Physiological Society Bethesda, MD.
